# Continuity and Admixture in the Last Five Millennia of Levantine History from Ancient Canaanite and Present-Day Lebanese Genome Sequences

**DOI:** 10.1016/j.ajhg.2017.06.013

**Published:** 2017-07-27

**Authors:** Marc Haber, Claude Doumet-Serhal, Christiana Scheib, Yali Xue, Petr Danecek, Massimo Mezzavilla, Sonia Youhanna, Rui Martiniano, Javier Prado-Martinez, Michał Szpak, Elizabeth Matisoo-Smith, Holger Schutkowski, Richard Mikulski, Pierre Zalloua, Toomas Kivisild, Chris Tyler-Smith

**Affiliations:** 1 Wellcome Trust Sanger Institute, Wellcome Genome Campus, Hinxton, Cambridgeshire CB10 1SA, UK; 2 The Sidon excavation, Saida, Lebanon; 3 Department of Archaeology and Anthropology, University of Cambridge, Cambridge CB2 1QH, UK; 4 Institute of Physiology, University of Zurich, Winterthurerstrasse 190, 8057 Zürich, Switzerland; 5 Department of Anatomy, University of Otago, Dunedin 9054, New Zealand; 6 Department of Archaeology, Anthropology, and Forensic Science, Bournemouth University, Talbot Campus, Poole BH12 5BB, UK; 7 The Lebanese American University, Chouran, Beirut 1102 2801, Lebanon; 8 Harvard T.H. Chan School of Public Health, Boston, MA 02115, USA

**Keywords:** aDNA, Bronze Age, whole-genome sequences, Near East, Lebanon, Sidon, Phoenicians, population genetic history

## Abstract

The Canaanites inhabited the Levant region during the Bronze Age and established a culture that became influential in the Near East and beyond. However, the Canaanites, unlike most other ancient Near Easterners of this period, left few surviving textual records and thus their origin and relationship to ancient and present-day populations remain unclear. In this study, we sequenced five whole genomes from ∼3,700-year-old individuals from the city of Sidon, a major Canaanite city-state on the Eastern Mediterranean coast. We also sequenced the genomes of 99 individuals from present-day Lebanon to catalog modern Levantine genetic diversity. We find that a Bronze Age Canaanite-related ancestry was widespread in the region, shared among urban populations inhabiting the coast (Sidon) and inland populations (Jordan) who likely lived in farming societies or were pastoral nomads. This Canaanite-related ancestry derived from mixture between local Neolithic populations and eastern migrants genetically related to Chalcolithic Iranians. We estimate, using linkage-disequilibrium decay patterns, that admixture occurred 6,600–3,550 years ago, coinciding with recorded massive population movements in Mesopotamia during the mid-Holocene. We show that present-day Lebanese derive most of their ancestry from a Canaanite-related population, which therefore implies substantial genetic continuity in the Levant since at least the Bronze Age. In addition, we find Eurasian ancestry in the Lebanese not present in Bronze Age or earlier Levantines. We estimate that this Eurasian ancestry arrived in the Levant around 3,750–2,170 years ago during a period of successive conquests by distant populations.

## Main Text

The Near East, including the Levant, has been central to human prehistory and history from the expansion out of Africa 50–60 thousand years ago (kya),^1^ through post-glacial expansions^2^ and the Neolithic transition 10 kya, to the historical period when Ancient Egyptians, Greeks, Phoenicians, Assyrians, Babylonians, Persians, Romans, and many others left their impact on the region.^3^ Aspects of the genetic history of the Levant have been inferred from present-day DNA,^4^, ^5^ but the more comprehensive analyses performed in Europe^6^, ^7^, ^8^, ^9^, ^10^, ^11^ have shown the limitations of relying on present-day information alone and highlighted the power of ancient DNA (aDNA) for addressing questions about population histories.^12^ Unfortunately, although the few aDNA results from the Levant available so far are sufficient to reveal how much its history differs from that of Europe,^13^ more work is needed to establish a thorough understanding of Levantine genetic history. Such work is hindered by the hot and sometimes wet environment,^12^, ^13^ but improved aDNA technologies including use of the petrous bone as a source of DNA^14^ and the rich archaeological remains available encouraged us to further explore the potential of aDNA in this region. Here, we present genome sequences from five Bronze Age Lebanese samples and show how they improve our understanding of the Levant’s history over the last five millennia.

During the Bronze Age in the Levant, around 3–4 kya, a distinctive culture emerged as a Semitic-speaking people known as the Canaanites. The Canaanites inhabited an area bounded by Anatolia to the north, Mesopotamia to the east, and Egypt to the south, with access to Cyprus and the Aegean through the Mediterranean. Thus the Canaanites were at the center of emerging Bronze Age civilizations and became politically and culturally influential.^15^ They were later known to the ancient Greeks as the Phoenicians who, 2.3–3.5 kya, colonized territories throughout the Mediterranean reaching as far as the Iberian Peninsula.^16^ However, for uncertain reasons but perhaps related to the use of papyrus instead of clay for documentation, few textual records have survived from the Canaanites themselves and most of their history known today has been reconstructed from ancient Egyptian and Greek records, the Hebrew Bible, and archaeological excavations.^15^ Many uncertainties still surround the origin of the Canaanites. Ancient Greek historians believed their homeland was located in the region of the Persian Gulf,^16^, ^17^ but modern researchers tend to reject this hypothesis because of archaeological and historical evidence of population continuity through successive millennia in the Levant. The Canaanite culture is alternatively thought to have developed from local Chalcolithic people who were themselves derived from people who settled in farming villages 9–10 kya during the Neolithic period.^15^ Uncertainties also surround the fate of the Canaanites: the Bible reports the destruction of the Canaanite cities and the annihilation of its people; if true, the Canaanites could not have directly contributed genetically to present-day populations. However, no archaeological evidence has so far been found to support widespread destruction of Canaanite cities between the Bronze and Iron Ages: cities on the Levant coast such as Sidon and Tyre show continuity of occupation until the present day.

aDNA research has the potential to resolve many questions related to the history of the Canaanites, including their place of origin and fate. Here, we sampled the petrous portion of temporal bones belonging to five ancient individuals dated to between 3,750 and 3,650 years ago (ya) from Sidon, which was a major Canaanite city-state during this period (Figures S1 and S2). We extracted DNA and built double-stranded libraries according to published protocols without uracil-DNA glycosylase treatment.^18^, ^19^, ^20^, ^21^ We sequenced the libraries on an Illumina HiSeq 2500 using 2× 75 bp reads and processed the sequences using the PALEOMIX pipeline.^22^ We retained reads ≥30 bp and collapsed pairs with minimum overlap of 15 bp, allowing a mismatch rate of 0.06 between the pairs. We mapped the merged sequences to the *hs37d5* reference sequence, removed duplicates, removed two bases from the ends of each read, and randomly sampled a single sequence with a minimum quality of ≥20 to represent each SNP. We obtained a genomic coverage of 0.4–2.3× and a mitochondrial DNA (mtDNA) genome coverage of 53–164× (Table 1). Y chromosome genotypes were jointly called across males from the 1000 Genomes Project, present-day Lebanese, and two identified Canaanite males using FreeBayes v.0.9.18.^23^ A maximum likelihood phylogeny was inferred using RAxML v.8.2.10^24^ and visualized using iTOL v.3.5.3.^25^ In order to assess ancient DNA authenticity, we estimated mtDNA and X chromosome contamination^26^, ^27^, ^28^ (Table S1) and restricted some analyses to sequences with aDNA damage patterns^29^, ^30^ (Figures S3 and S4), demonstrating that the sequence data we present are endogenous and minimally contaminated.

**Table 1:** Samples Analyzed in This Study

**ENA Number**	**Burial Number**	**Time Years Ago**	**Mapped Reads**^a^	**Mapped Read %**	**Coverage Genomic**	**Coverage MT**	**Sex**^b^	**MT Haplogroup**	**Y Haplogroup**
ERS1790733	54	3,700^c^	69,084,826	6.24	1.19	110	M	N1a3a	J1-P58
ERS1790732	63	3,650^d^	98,293,308	9.20	1.69	109	M	HV1b1	J2-M12
ERS1790730	65	3,650^d^	73,701,096	7.57	1.24	124	F	K1a2	–
ERS1790731	75	3,750^d^	128,355,897	15.48	2.32	164	F	R2	–
ERS1790729	46	3,750^d^	23,323,399	2.64	0.40	53	F	H1bc	–

a Excluding PCR duplicates

b Genetically determined

c Radiocarbon date

d Archaeological date

Additionally, we sequenced whole genomes of 99 present-day Lebanese individuals with informed consent to ∼8× coverage on an Illumina HiSeq 2500 using 2× 100 bp reads in a study approved by The Wellcome Trust Sanger Institute’s Human Materials and Data Management Committee (13/010 and 14/072). We merged the low-coverage Lebanese data with four high-coverage (30×) Lebanese samples,^31^ 1000 Genomes Project phase 3 CEU, YRI, and CHB populations,^32^ and sequence data previously published from regional populations (Egyptians, Ethiopians, and Greeks).^1^, ^31^ Raw calls were generated using bcftools (bcftools mpileup -C50 -pm3 -F0.2 -d10000 | bcftools call -mv, version 1.2-239-g8749475) and filtered to include only SNPs with the minimum of two alternate alleles in at least one population and site quality larger than ten; we excluded sites with a minimum per-population HWE and total HWE less than 0.01^33^ and sites within 3 bp of an indel. The filtered calls were then pre-phased using shapeit (v.2.r790)^34^ and their genotypes refined using beagle (v.4.1).^35^ We have previously described the genetic structure in the Lebanese population using array data from ∼1,300 individuals.^4^ A principal component analysis (PCA) using the 99 sequenced present-day individuals show that they capture the previously described genetic diversity with distinct clusters reflecting the different cultural groups in Lebanon today (Figure S5).

**Figure 1:**
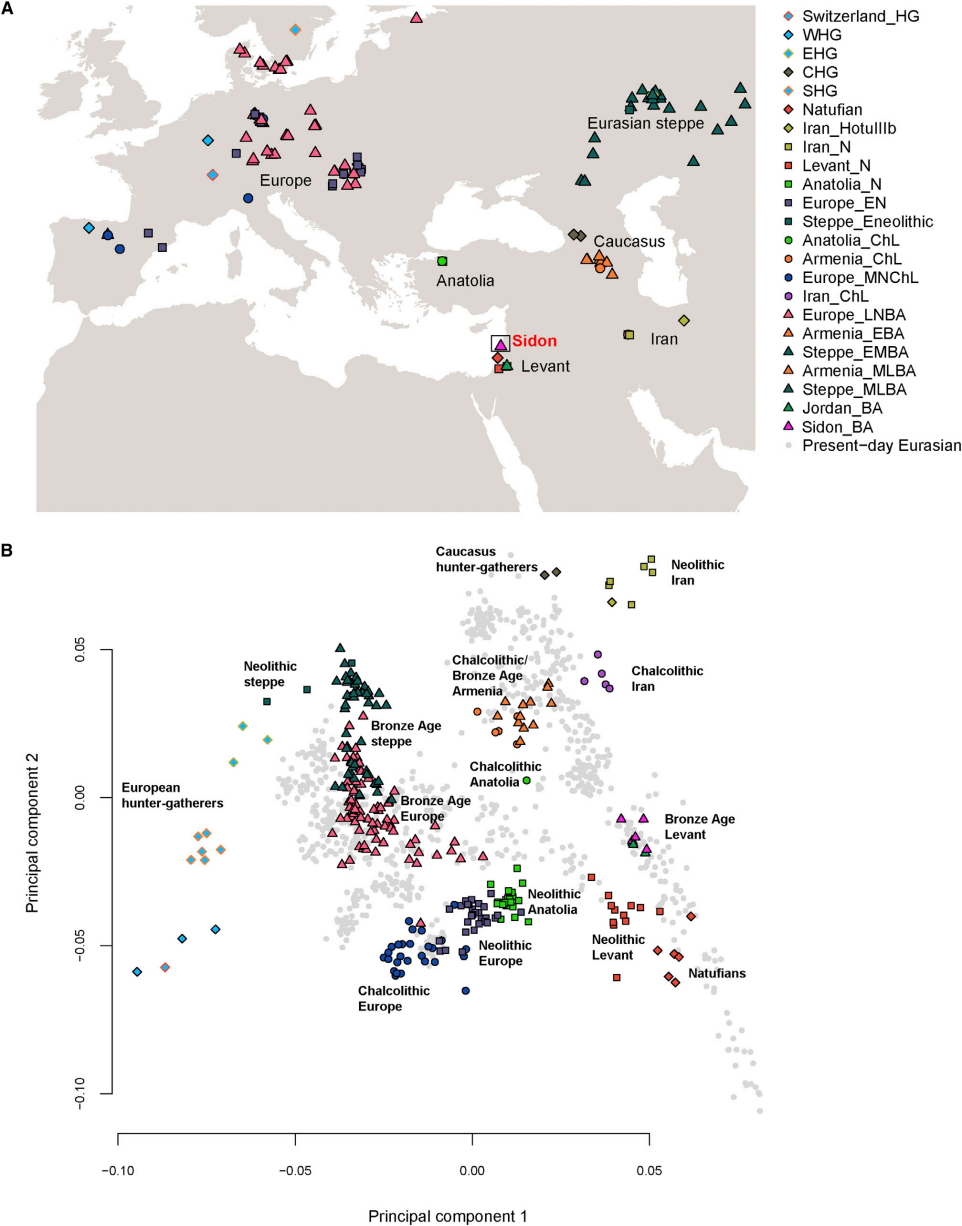
Population Locations and Genetic Structure (A) The map shows the location of the newly sequenced Bronze Age Sidon samples (pink triangle labeled with red text), as well as the locations of published ancient samples used as comparative data in this study. (B) PCA of ancient Eurasian samples (colored shapes) projected using eigenvectors from present-day Eurasian populations (gray points).

We combined our ancient and modern samples with previously published ancient data^6^, ^7^, ^8^, ^9^, ^10^, ^11^, ^13^, ^36^, ^37^, ^38^ (Figure 1A) resulting in a dataset of 389 individuals and 1,046,317 SNPs when ancient and Lebanese samples were analyzed, and 546,891 SNPs when 2,583 modern samples from the Human Origins genotype data were included in the analysis (i.e., the small dataset was used only when a modern population other than the Lebanese was included in the test).^9^, ^39^ A pooled Lebanese sequence dataset (99 low coverage plus 4 high coverage) was used in all analyses except for the PCA and ADMIXTURE where a subset of 15 randomly selected individuals (5 from each group described in Figure S5) was used to avoid sample size bias. The ancient samples were grouped following the labels assigned by Lazaridis et al.^13^ on the basis of archaeological culture, chronology, and genetic clustering. We used this dataset to shed light on the genetic history of the Canaanites, resolving their relationship to other ancient populations and assessing their genetic contribution to present-day populations.

We first explored our dataset using PCA^40^ on present-day West Eurasian (including Levantine) populations and projected the ancient samples onto this plot (Figures 1B and S6). The Bronze Age Sidon samples (Sidon_BA) overlap with present-day Levantines and were positioned between the ancient Levantines (Natufians/Neolithic) and ancient Iranians (Neolithic/Chalcolithic). The overlap between the Bronze Age and present-day Levantines suggests a degree of genetic continuity in the region. We explored this further by computing the statistic *f4*(Lebanese, present-day Near Easterner; Sidon_BA, Chimpanzee) using *qpDstat*^39^ (with parameter f4mode: YES) and found that Sidon_BA shared more alleles with the Lebanese than with most other present-day Levantines (Figure S7), supporting local population continuity as observed in Sidon’s archaeological records. When we substituted present-day Near Easterners with a panel of 150 present-day populations available in the Human Origins dataset, we found that only Sardinians and Italian_North shared significantly more alleles with Sidon_BA compared with the Lebanese (Figure S8). Sardinians are known to have retained a large proportion of ancestry from Early European farmers (EEFs) and therefore the increased affinity to Sidon_BA could be related to a shared Neolithic ancestry. We computed *f4*(Lebanese, Sardinian/Italian_North; Sidon_BA, Levant_N) and found no evidence of increased affinity of Sardinians or Italian_North to Sidon_BA after the Neolithic (both Z-scores are positive). We next wanted to explore whether the increased affinity of Sidon_BA to the Lebanese could also be observed when analyzing functionally important regions of the genome that are less susceptible to genetic drift. Our sequence data allowed us to scan loci linked to phenotypic traits and loci previously identified as functional variants in the Lebanese and other Levantines.^41^, ^42^, ^43^ Using a list of 84 such variants (Table S2), we estimated the allele frequency (AF) in Sidon_BA using ANGSD^26^ based on a method from Li et al.^44^ and calculated Pearson pairwise correlation coefficients between AF in Sidon_BA and AF in Africans, Europeans, Asians,^32^ and Lebanese. We found a high significant correlation between Sidon_BA and the Lebanese (r = 0.74; 95% CI = 0.63–0.82; p value = 8.168 × 10^−16^) and lower correlations between Sidon_BA and Europeans (r = 0.56), Africans (r = 0.55), and Asians (r = 0.53) (Figure S9). These results support population continuity in the region and suggest that several present-day genetic disorders might stem from risk alleles that were already present in the Bronze Age population. In addition, SNPs associated with phenotypic traits show that Sidon_BA and the Lebanese had comparable skin, hair, and eye colors (in general: light intermediate skin pigmentation, brown eyes, and dark hair) with similar frequencies of the underlying causal variants in *SLC24A5* and *HERC2*, but with Sidon_BA probably having darker skin than Lebanese today from variants in *SLC45A2* resulting in darker pigmentation (Table S2).

**Figure 2:**
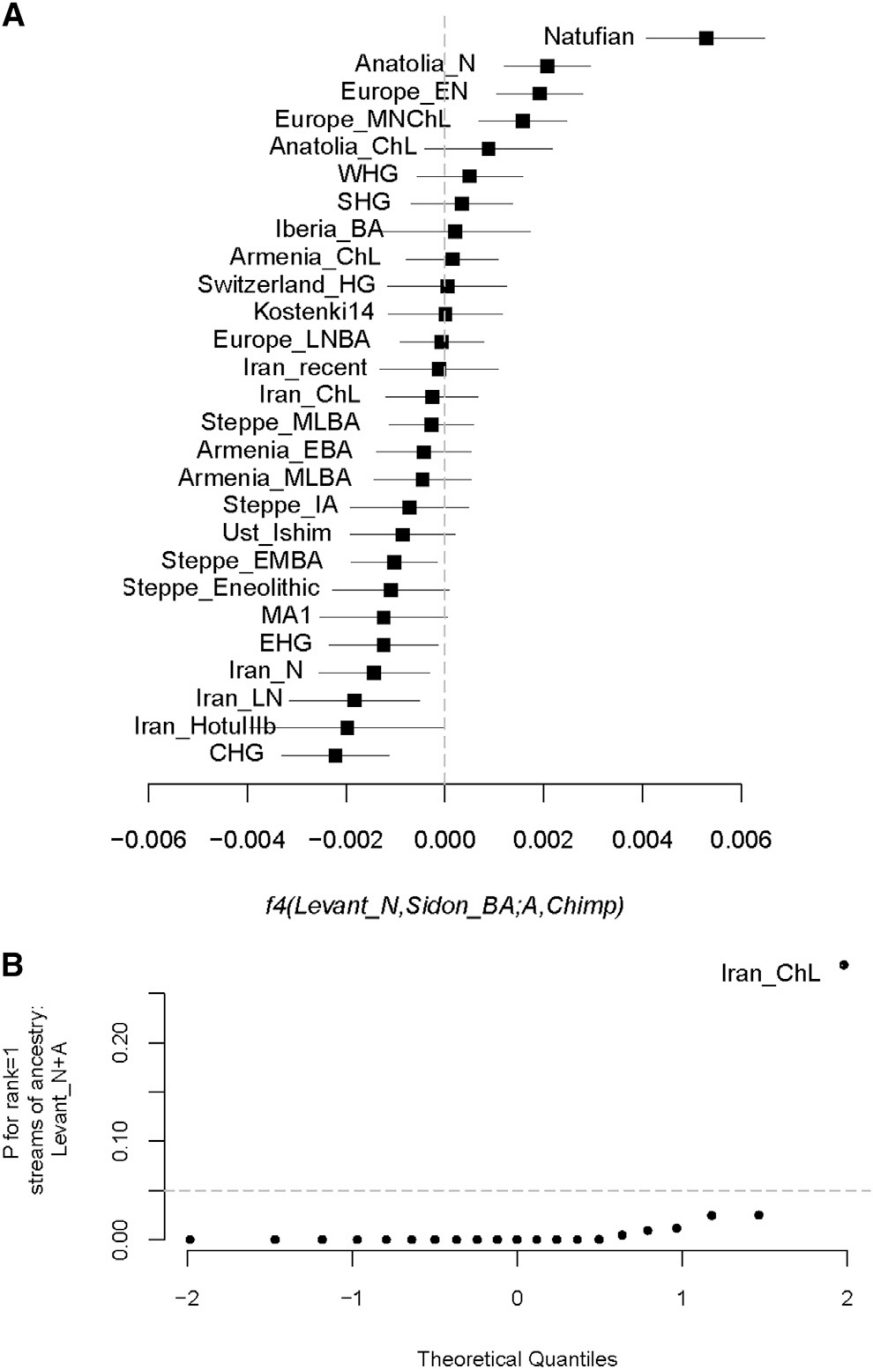
Admixture in Bronze Age Levantine Populations (A) The statistic *f4*(Levant_N, Sidon_BA; Ancient Eurasian, Chimpanzee) is most negative for ancient populations from the Caucasus and Iran, suggesting an increase in ancestry related to these populations in Sidon after the Neolithic period. The plot shows the estimated statistic value and ±3 standard errors. (B) Modeling Sidon as mixture between Neolithic Levant and an ancient Eurasian population shows that Chalcolithic Iran fits the model best when using a large number of outgroups: Ust_Ishim, Kostenki14, MA1, Han, Papuan, Ami, Chukchi, Karitiana, Mbuti, Switzerland_HG, EHG, WHG, and CHG. Sidon_BA can then be modeled using *qpAdm* as 0.484 ± 0.042 Levant_N and 0.516 ± 0.042 Iran_ChL.

The PCA shows that Sidon_BA clusters with three individuals from Early Bronze Age Jordan (Jordan_BA) found in a cave above the Neolithic site of ‘Ain Ghazal and probably associated with an Early Bronze Age village close to the site.^13^ This suggests that people from the highly differentiated urban culture on the Levant coast and inland people with different modes of subsistence were nevertheless genetically similar, supporting previous reports that the different cultural groups who inhabited the Levant during the Bronze Age, such as the Ammonites, Moabites, Israelites, and Phoenicians, each achieved their own cultural identities but all shared a common genetic and ethnic root with Canaanites.^15^ Lazaridis et al.^13^ reported that Jordan_BA can be modeled as mixture of Neolithic Levant (Levant_N) and Chalcolithic Iran (Iran_ChL). We computed the statistic *f4*(Levant_N, Sidon_BA; Ancient Eurasian, Chimpanzee) and found that populations from the Caucasus and ancient Iran shared more alleles with Sidon_BA than with Neolithic Levant (Figure 2A and S10). We then used *qpAdm*^8^ (with parameter allsnps: YES) to test whether Sidon_BA can be modeled as mixture of Levant_N and any other ancient population in the dataset and found good support for the model of Sidon_BA being a mixture of Levant_N (48.4% ± 4.2%) and Iran_ChL (51.6% ± 4.2%) (Figure 2B; Table S3).

In addition, the two Sidon_BA males carried the Y-chromosome haplogroups^45^ J-P58 (J1a2b) and J-M12 (J2b) (Tables 1 and S4; Figure S11), both common male lineages in the Near East today. Haplogroup J-P58 is frequent in the Arabian peninsula with proposed origins in the Zagros/Taurus mountain region.^46^ It forms the vast majority of the Y chromosomes in southwestern Mesopotamia and reaches particularly high frequencies (74.1%) in Marsh Arabs in Iraq.^47^ On the other hand, haplogroup J-M12 is widespread at low frequency from the Balkans to India and the Himalayas, with Albanians having the highest proportions (14.3%).^48^ We compiled frequencies of Y-chromosome haplogroups in this geographical area and their changes over time in a dataset of ancient and modern Levantine populations (Figure S12), and note, similarly to Lazaridis et al.,^13^ that haplogroup J was absent in all Natufian and Neolithic Levant male individuals examined thus far, but emerged during the Bronze Age in Lebanon and Jordan along with ancestry related to Iran_ChL. All five Sidon_BA individuals had different mitochondrial DNA haplotypes^49^ (Table 1), belonging to paragroups common in present-day Lebanon and nearby regions (Table S5) but with additional derived variants not observed in our present-day Lebanese dataset.

We next sought to estimate the time when the Iran_ChL-related ancestry penetrated the Levant. Our results support genetic continuity since the Bronze Age and thus our large dataset of present-day Lebanese provided an opportunity to explore the admixture time using admixture-induced linkage disequilibrium (LD) decay. Using ALDER^50^ (with mindis: 0.005), we set the Lebanese as the admixed test population and Natufians, Levant_N, Sidon_BA, Iran_N, and Iran_ChL as reference populations. To account for the small number of individuals in the reference populations and the limited number of SNPs in the dataset, we took a lenient minimum Z-score = 2 to be suggestive of admixture. The most significant result was for mixture of Levant_N and Iran_ChL (p = 0.013) around 181 ± 54 generations ago, or ∼5,000 ± 1,500 ya assuming a generation time of 28 years (Figure S13A). This admixture time, based entirely on genetic data, fits the known ages of the samples based on archaeological data since it falls between the dates of Sidon_BA (3,650–3,750 ya) and Iran_ChL (6,500–5,500 ya). The admixture time also overlaps with the rise and fall of the Akkadian Empire which controlled the region from Iran to the Levant between ∼4.4 and 4.2 kya. The Akkadian collapse is argued to have been the result of a widespread aridification event around 4,200 ya.^51^, ^52^ Archaeological evidence in this period documents large-scale influxes of refugees from Northern Mesopotamia toward the south, where cities and villages became overpopulated.^53^ Our confidence intervals for the admixture dates are wide and therefore the historical links suggested here should be considered with caution. Future sampling of ancient DNA from northern Syria and Iraq will reveal whether these populations carried the Iran_ChL-related ancestry and also provide a better understanding of the origin of the eastern migrants and the time when they arrived in the Levant.

**Figure 3:**
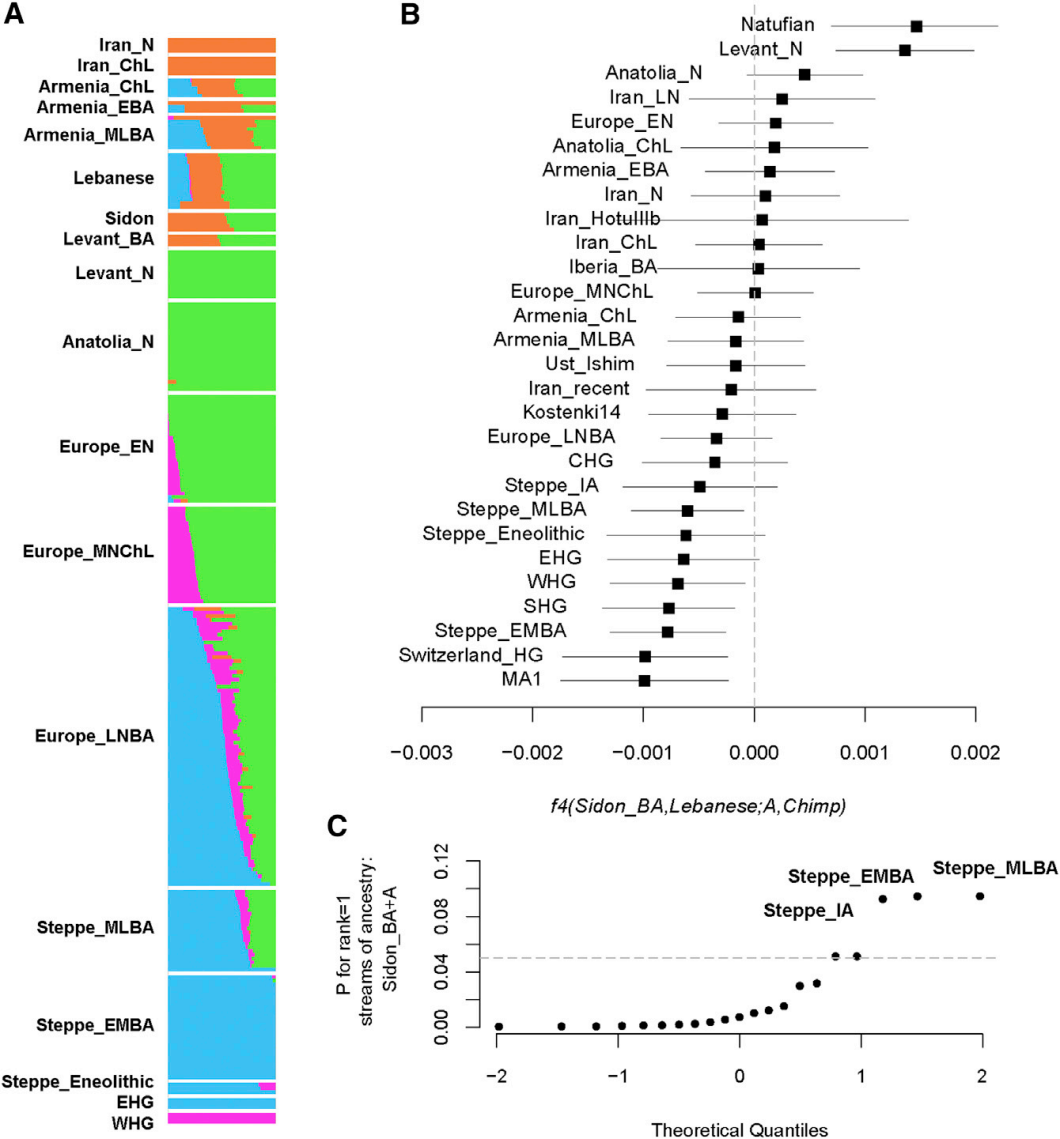
Admixture in Present-Day Levantine Populations (A) Supervised ADMIXTURE using Levant_N, Iran_N, EHG, and WHG as reference populations. A Eurasian ancestry found in Eastern hunter-gatherers and the steppe Bronze Age appears in present-day Levantines after the Bronze Age. (B) The statistic *f4*(Sidon_BA, Lebanese; Ancient Eurasian, Chimpanzee) confirms the ADMIXTURE results and is most negative for populations from the steppe and Eurasian hunter-gatherers. We show the estimated statistic value and ±3 standard errors. (C) Present-day Lebanese can be modeled as mixture between Bronze Age Sidon and a steppe population. The model with mix proportions 0.932 ± 0.016 Sidon_BA and 0.068 ± 0.016 steppe_EMBA for Lebanese is supported with the lowest SE.

Although *f4* tests showed that present-day Lebanese share significantly more alleles with Sidon_BA than other Near Eastern populations do, indicating genetic continuity, we failed to model the present-day Lebanese using streams of ancestry coming only from Levant_N and Iran_ChL (*qpAdm* rank1 p = 8.36 × 10^−7^), in contrast to our success with Sidon_BA. We therefore further explored our dataset by running ADMIXTURE^54^ in a supervised mode using Western hunter-gatherers (WHG), Eastern hunter-gatherers (EHG), Levant_N, and Iran_N as reference populations. These four populations have been previously^13^ found to contribute genetically to most West Eurasians. The ADMIXTURE results replicate the findings from *qpAdm* for Sidon_BA and show mixture of Levant_N and ancient Iranian populations (Figure 3A). However, the present-day Lebanese, in addition to their Levant_N and ancient Iranian ancestry, have a component (11%–22%) related to EHG and Steppe populations not found in Bronze Age populations (Figure 3A). We confirm the presence of this ancestry in the Lebanese by testing *f4*(Sidon_BA, Lebanese; Ancient Eurasian, Chimpanzee) and find that Eurasian hunter-gatherers and Steppe populations share more alleles with the Lebanese than with Sidon_BA (Figures 3B and S14). We next tested a model of the present-day Lebanese as a mixture of Sidon_BA and any other ancient Eurasian population using *qpAdm*. We found that the Lebanese can be best modeled as Sidon_BA 93% ± 1.6% and a Steppe Bronze Age population 7% ± 1.6% (Figure 3C; Table S6). To estimate the time when the Steppe ancestry penetrated the Levant, we used, as above, LD-based inference and set the Lebanese as admixed test population with Natufians, Levant_N, Sidon_BA, Steppe_EMBA, and Steppe_MLBA as reference populations. We found support (p = 0.00017) for a mixture between Sidon_BA and Steppe_EMBA which has occurred around 2,950 ± 790 ya (Figure S13B). It is important to note here that Bronze Age Steppe populations used in the model need not be the actual ancestral mixing populations, and the admixture could have involved a population which was itself admixed with a Steppe-like ancestry population. The time period of this mixture overlaps with the decline of the Egyptian empire and its domination over the Levant, leading some of the coastal cities to thrive, including Sidon and Tyre, which established at this time a successful maritime trade network throughout the Mediterranean. The decline in Egypt’s power was also followed by a succession of conquests of the region by distant populations such as the Assyrians, Persians, and Macedonians, any or all of whom could have carried the Steppe-like ancestry observed here in the Levant after the Bronze Age.

**Figure 4:**
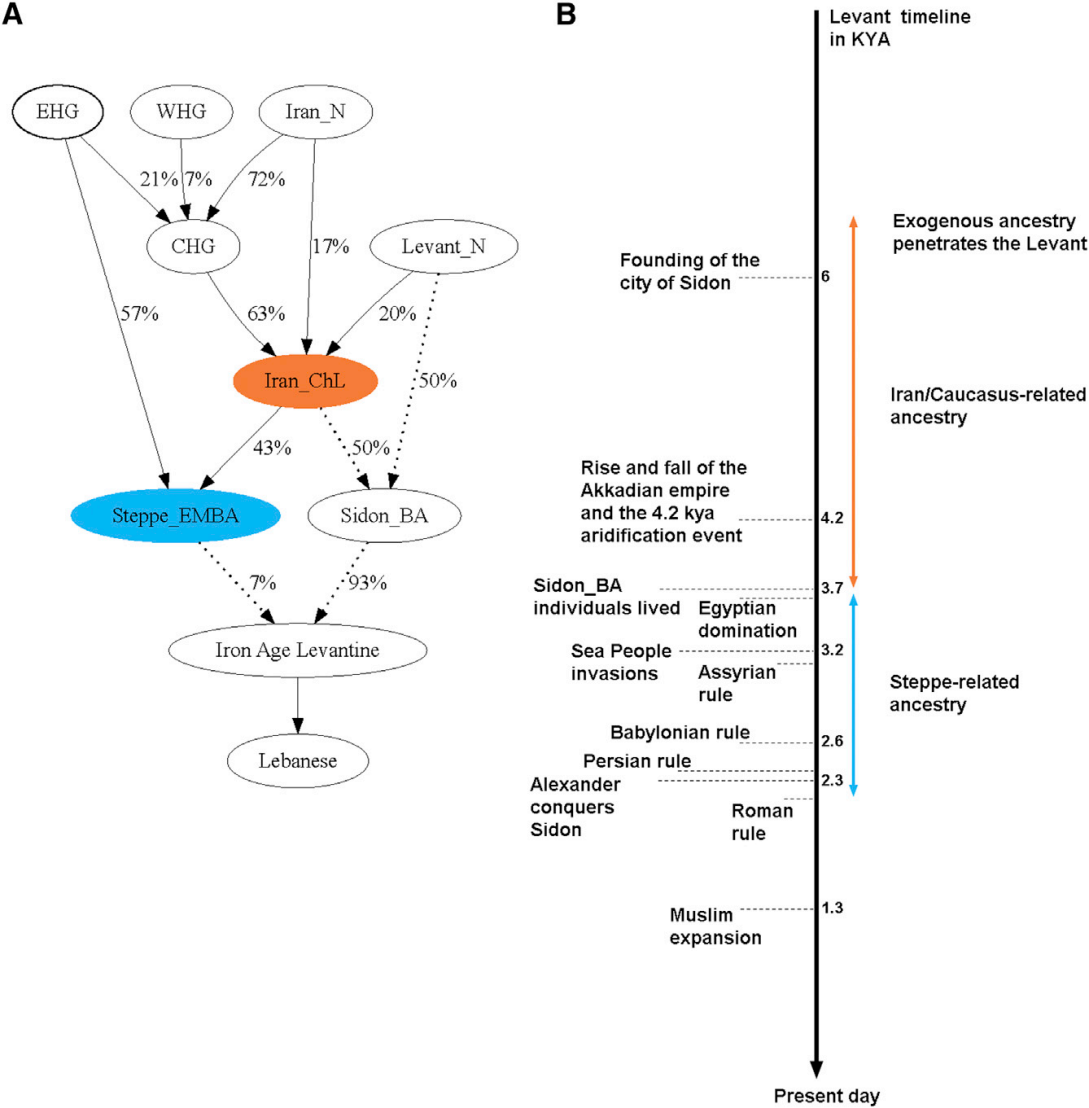
Genetic History of the Levant (A) A model of population relationships which fits the *qpAdm* results from Lazaridis et al.^13^ (solid arrows) and this study (dotted arrows). Percentages on arrows are the inferred admixture proportions. (B) Levant timeline of historical events with genetically inferred admixture dates shown as colored double-ended arrows with length representing the SE.

In this report we have analyzed ancient whole-genome sequence data from a Levantine civilization and provided insights into how the Bronze Age Canaanites were related to other ancient populations and how they have contributed genetically to present-day ones (Figure 4). Many of our inferences rely on the limited number of ancient samples available, and we are only just beginning to reconstruct a genetic history of the Levant or the Near East as thoroughly as that of Europeans who, in comparison, have been extensively sampled. In the future, it will be important to examine samples from the Chalcolithic/Early Bronze Age Near East to understand the events leading to admixture between local populations and the eastern migrants. It will also be important to analyze samples from the Iron Age to trace back the Steppe-like ancestry we find today in present-day Levantines. Our current results show that such studies are feasible.

## Accession Numbers

Sequencing data for 99 present-day Lebanese individuals reported in this paper are available through the European Genome-phenome Archive (EGA) under accession number EGA: EGAS00001002084. Raw sequencing reads for 5 ancient Canaanite individuals are available through the European Nucleotide Archive (ENA) under accession number ENA: PRJEB21330. Aligned sequences and genotypes can be obtained from the corresponding authors.

## Web Resources

European Genome-phenome Archive (EGA), https://www.ebi.ac.uk/ega

European Nucleotide Archive (ENA), http://www.ebi.ac.uk/ena

## Supplemental Data

Document S1. Figures S1–S14 and Tables S1–S6

Document S2. Article plus Supplemental Data
