# Supplementary material for: Continuity and Admixture in the Last Five Millennia of Levantine History from Ancient Canaanite and Present-Day Lebanese Genome Sequences
Source: Am J Hum Genet. 2017 Jul 27;101(2):274–82. doi: 10.1016/j.ajhg.2017.06.013 (PMC5544389; doi:10.1016/j.ajhg.2017.06.013)
Supplement: Document S1. Figures S1–S14 and Tables S1–S6 [file mmc1.pdf]

The American Journal of Human Genetics, Volume *101*

Supplemental Data

Continuity and Admixture in the Last Five Millennia of Levantine History from Ancient Canaanite and Present-Day Lebanese Genome Sequences

Marc Haber, Claude Doumet-Serhal, Christiana Scheib, Yali Xue, Petr Danecek, Massimo Mezzavilla, Sonia Youhanna, Rui Martiniano, Javier Prado-Martinez, Michał Szpak, Elizabeth Matisoo-Smith, Holger Schutkowski, Richard Mikulski, Pierre Zalloua, Toomas Kivisild, and Chris Tyler-Smith

Supplementary materials

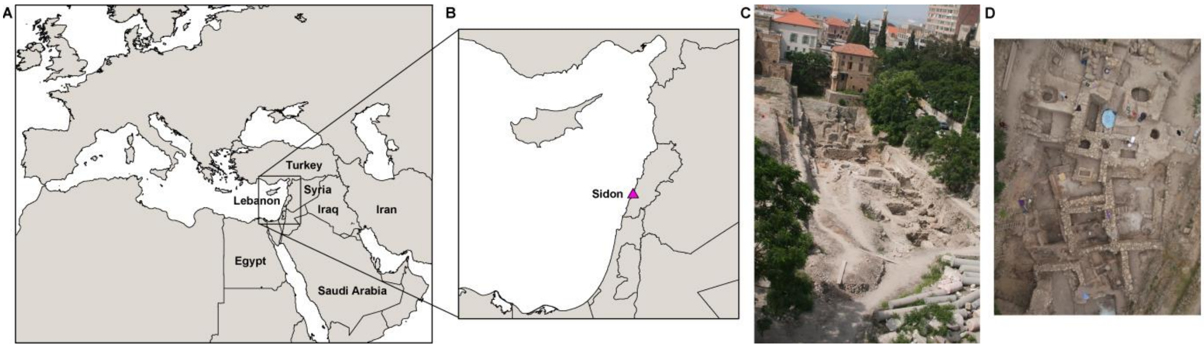

Figure S1. The Sidon excavation site. (A) Map shows the location of Lebanon with present-day political borders in the Near East. (B) A magnification showing the Levant region and the location of the city of Sidon. (C) Photo shows the Sidon excavation site which included the burials of individuals studied here. (D) Aerial view of excavation details from Season 12 (Bronze Age structures).

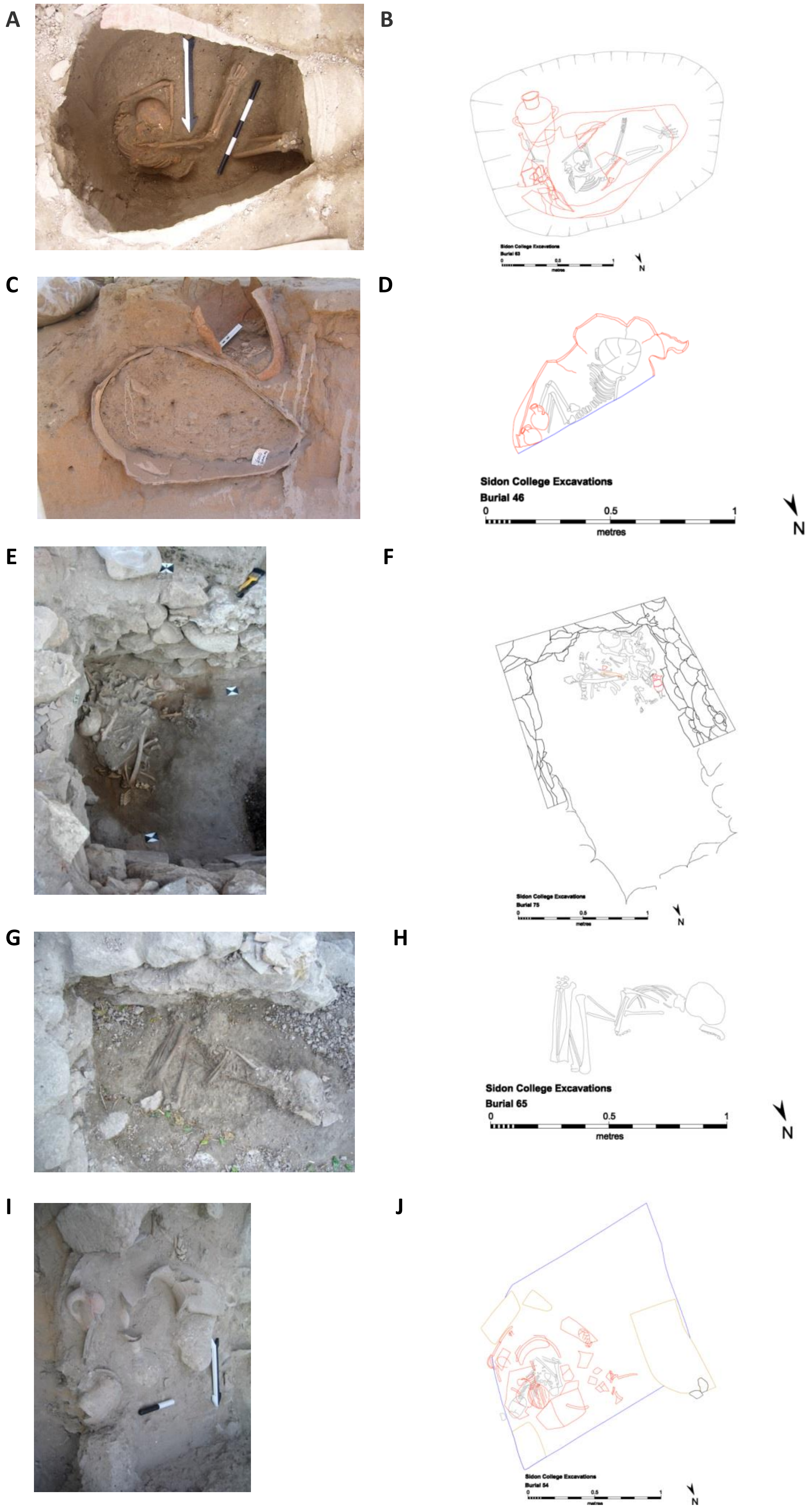

A
B
Sidon College Excavations
Burial 63
0
0.5
1
metres
N
C
D
Sidon College Excavations
Burial 46
0
0.5
1
metres
N
E
F
Sidon College Excavations
Burial 75
0
0.5
1
metres
N
G
H
Sidon College Excavations
Burial 65
0
0.5
1
metres
N
I
J
Sidon College Excavations
Burial 54
0
0.5
1
metres
N

Figure S2. Burials of individuals analysed in this study. (A) and (B) burial 63: Stratum 6 Middle Bronze Age II B about 1600 BC. Consisted of an unusually large burial containing the remains of at least two individuals. (C) and (D) burial 46: Intermediate Middle Bronze Age II A-B about 1750 BC. This burial consisted of a jar burial containing an infant. (E) and (F) burial 75: Intermediate Middle Bronze Age II A-B about 1750 BC. It contained the commingled remains of 3 individuals. (G) and (H) burial 65: Stratum 6 Middle Bronze Age II B about 1600 BC. This burial consisted of a simple unlined burial of a single sub adult individual. (I) and (J) burial 54: Middle Bronze Age II B around about 1650 BC. This jar consisted of the burial of a sub adult, probably 8-12 years old.

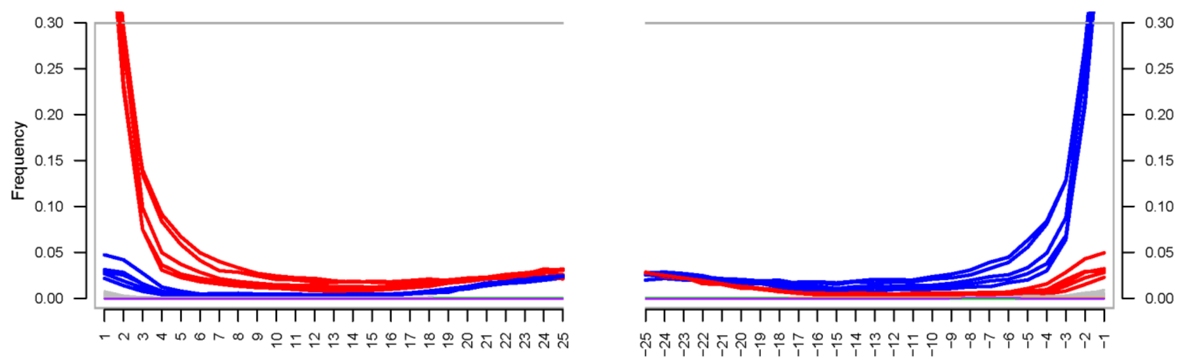

Figure S3. Post-mortem damage patterns. Base substitutions C>T from the 5" (left) and G>A from the 3" end (right) for all Sidon samples show patterns typical of ancient DNA damage.

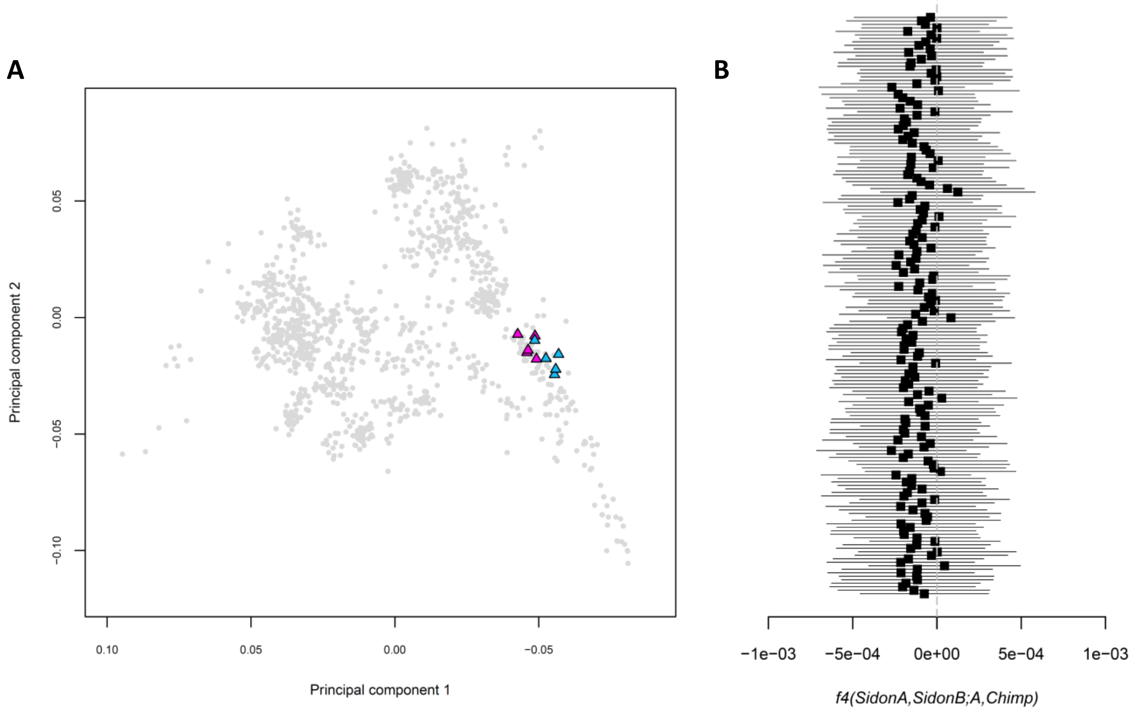

Figure S4. Testing for contamination using sequences with evidence of post-mortem damage (PMD). (A) PCA is similar to Figure 1 but additionally includes the ancient individuals represented only by sequences with a PMD score of at least 3 (blue triangles) to remove potentially contaminating sequences from present-day individuals. The samples overlap samples with no damage-restriction (pink triangles). (B) *f4* statistics testing if a modern population *A* in the dataset has excess affinity to samples with no damage-restriction (*SidonB*) compared with *SidonA* represented only by sequences with a PMD score >3. Tests for all modern populations do not significantly deviate from zero. The PCA and *f4* statistics suggest the Sidon DNA samples are authentic and minimally contaminated.

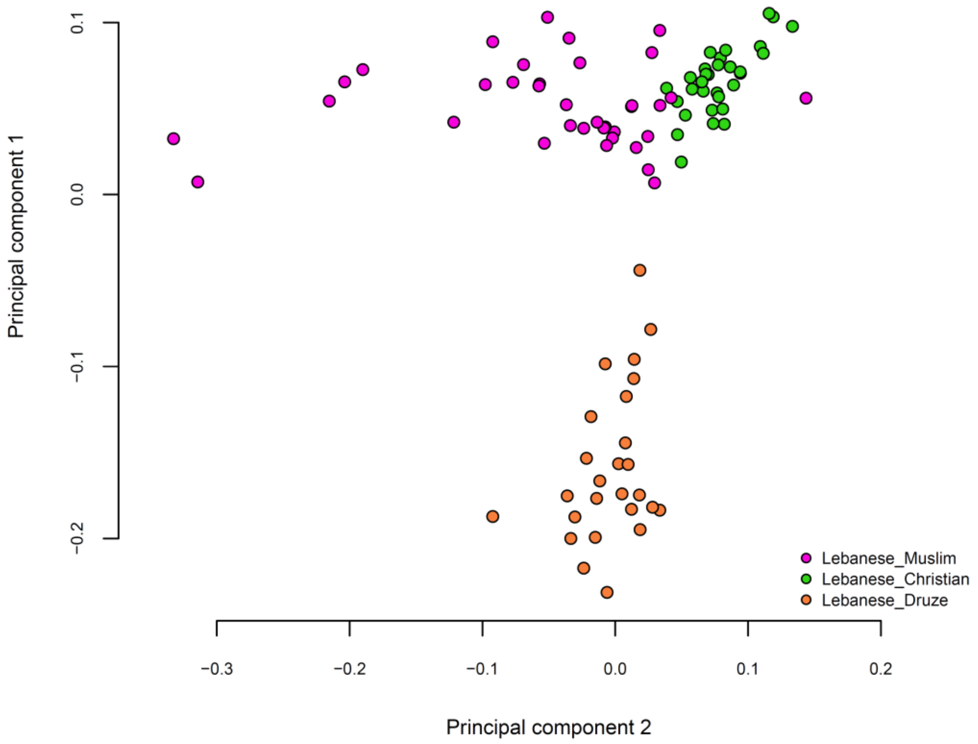

Figure S5. Genetic structure in present-day Lebanese. Principal components analysis of 99 present-day Lebanese sequenced in this study. We used the *smartpca* program of EIGENSOFT on SNPs overlapping with the 1.2M SNPs from Lazaridis et al. 2016[1] and set killr2: YES, r2thresh: 0.7, outliersigmathresh: 5. The genetic diversity of the sequenced Lebanese captures the previously-described genetic diversity in Lebanon[2] using a set with a larger sample size (1341) and array data.

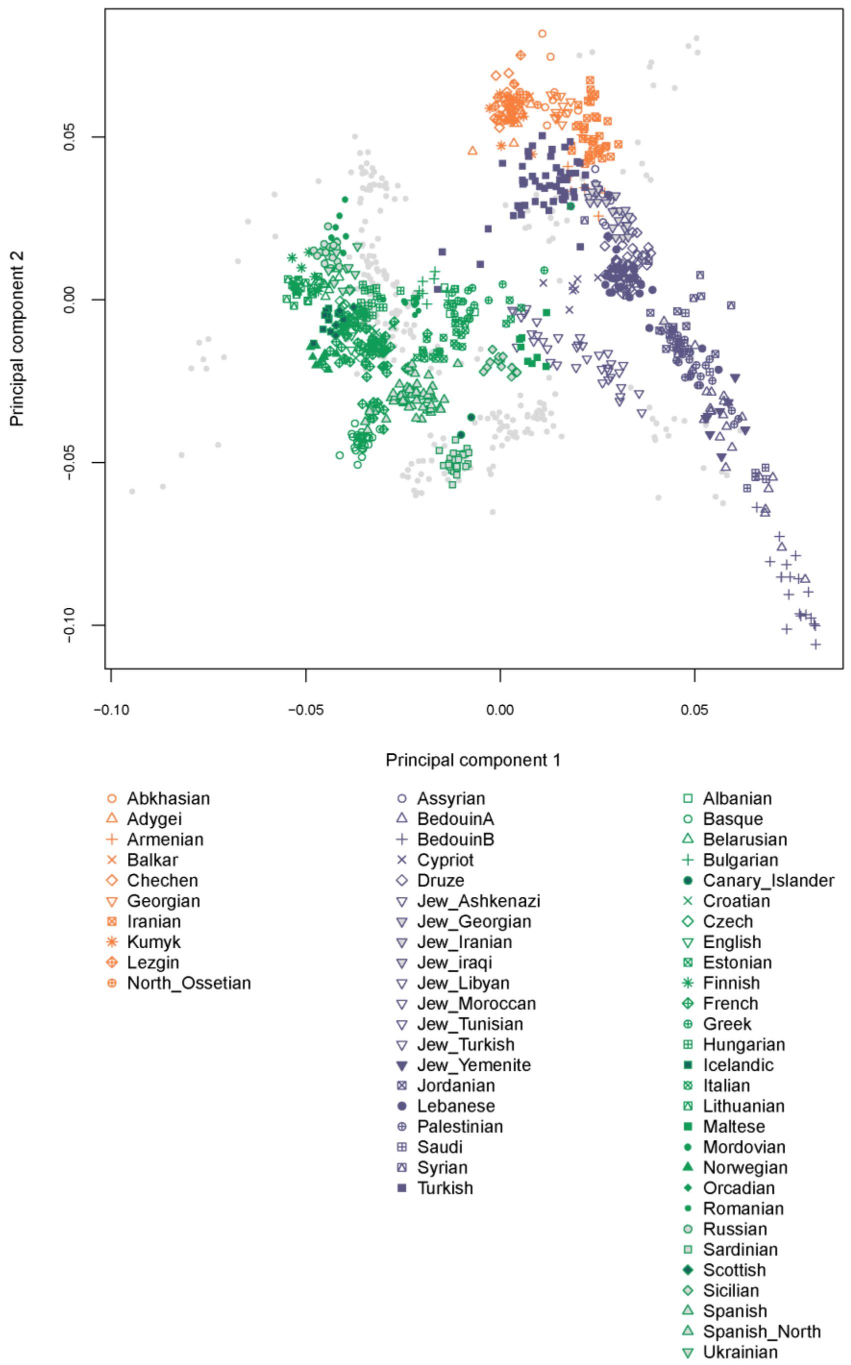

Figure S6. PCA showing the position of present-day populations. The gray points represent the projected ancient samples shown in Figure1.

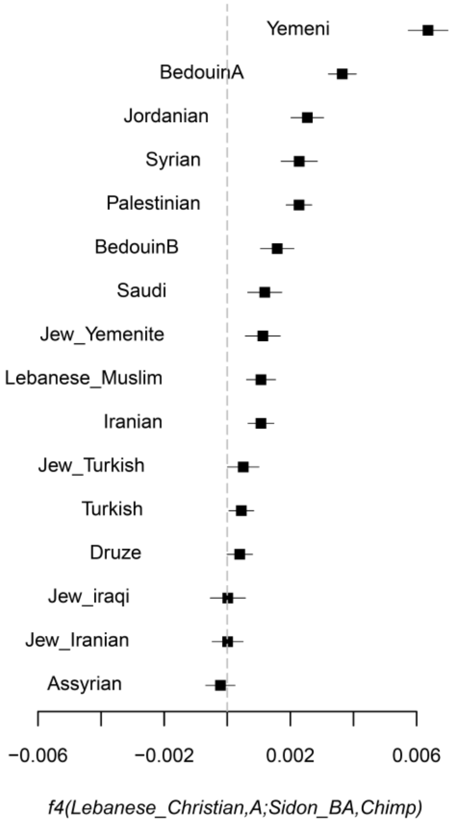

Figure S7. Testing Sidon_BA affinity to present-day Near Easterners. The statistic *f4*(Lebanese_Christian, *Test*; Sidon_BA, Chimpanzee) is significantly positive for most populations when *Test* is a present-day Near Easterner, indicating Sidon_BA shares more alleles with the Lebanese than with other Near Easterners. We chose Lebanese_Christian to represent present-day Lebanese in this test as this population has been shown to be relatively isolated and had no significant admixture in recent times with neighbouring populations. In this and following figures we plot the *f4* statistic value and ±3 standard errors.

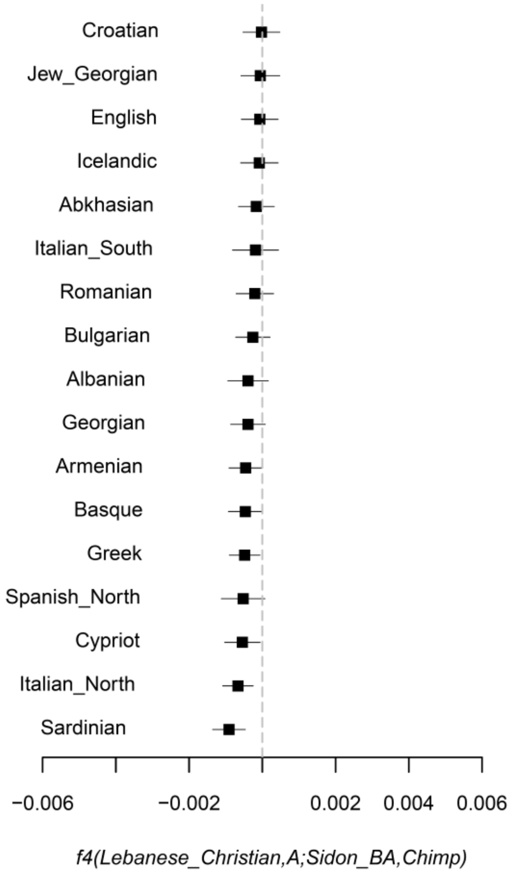

Figure S8. Testing Sidon_BA affinity to worldwide populations. The plot shows all the negative statistics from *f4*(Lebanese_Christian, *Test*; Sidon_BA, Chimpanzee) when *Test* is one of the 150 present-day populations available in the Human Origins dataset.

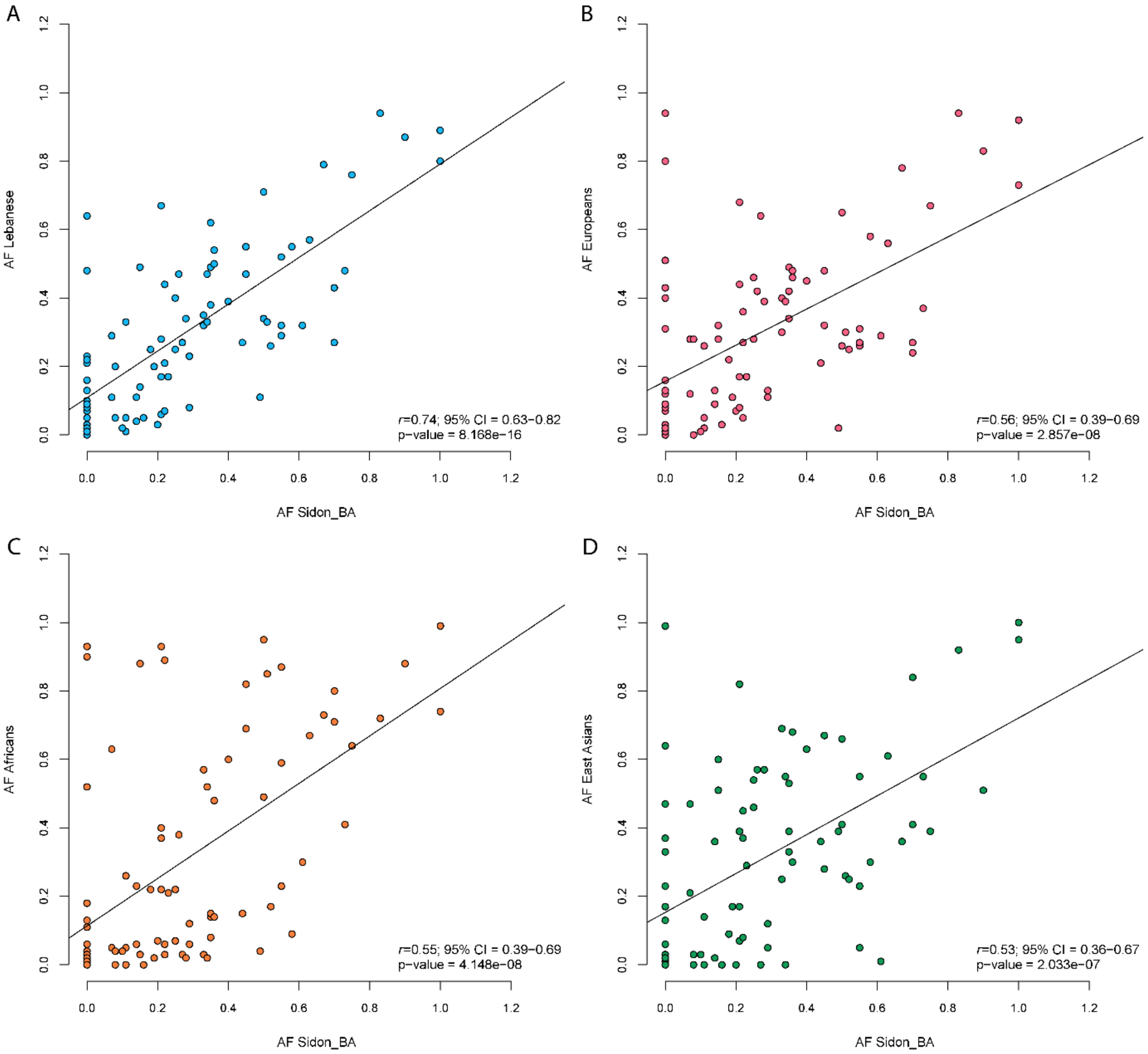

Figure S9. Correlation between the frequencies of the putative functional variants in Sidon_BA and present-day populations: (A) Lebanese (B) Europeans (C) Africans and (D) East Asians.

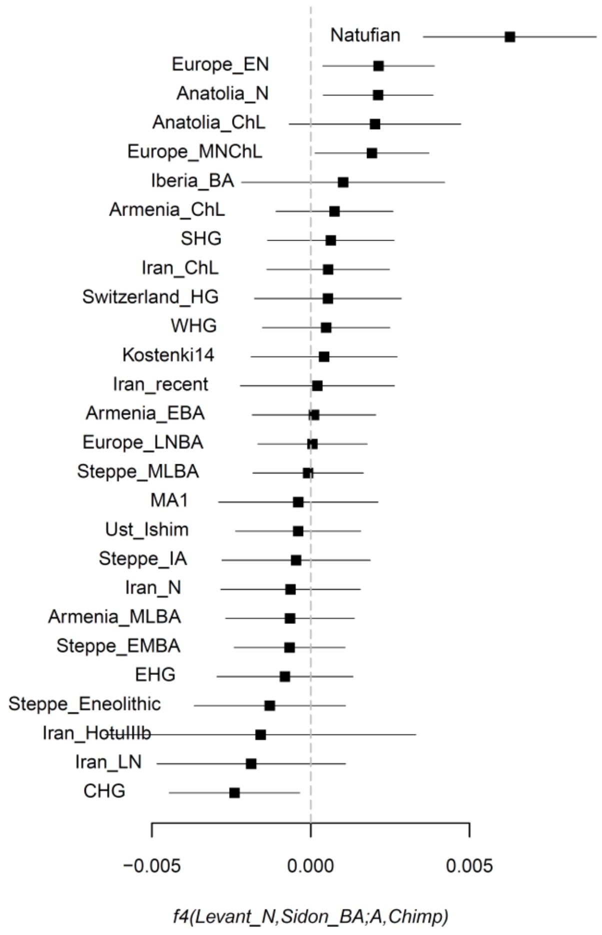

Figure S10. Admixture in Bronze Age Levantine populations (transversions only). We replicate the statistic *f4*(Levant_N, Sidon_BA; Ancient Eurasian, Chimpanzee) using ~120,000 transversions and get results qualitatively similar to the results from using all variants.

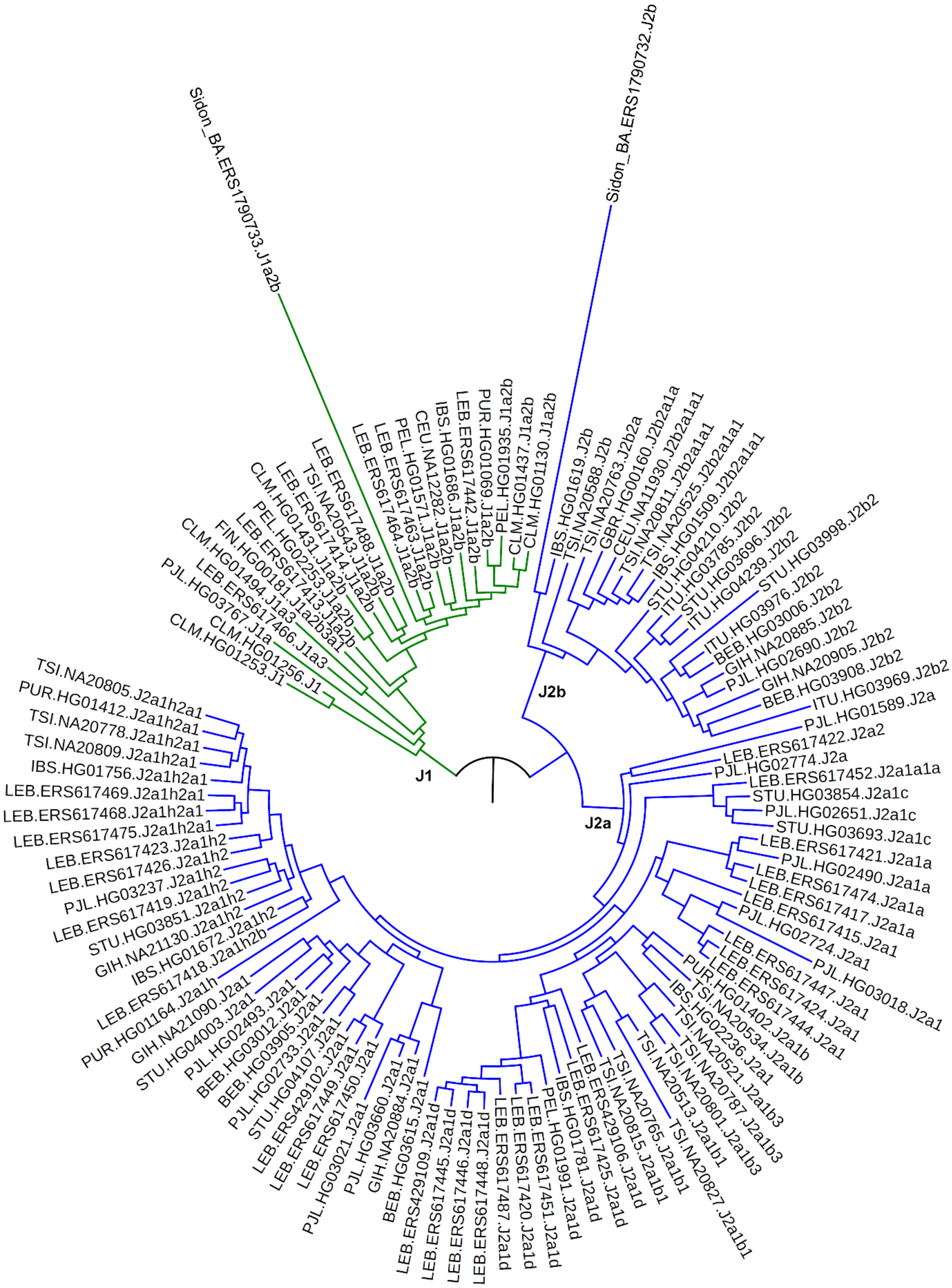

Figure S11. Y-chromosome J lineage relationship tree. A maximum likelihood phylogeny of Y chromosomes belonging to haplogroup J inferred using RAxML on males from the 1000 Genomes Project (76), present-day Lebanese (35), and Sidon_BA (2). Tip labels show population and sample name as well as haplogroup identified by yHaplo using ISOGG 2016.01.04 annotations.

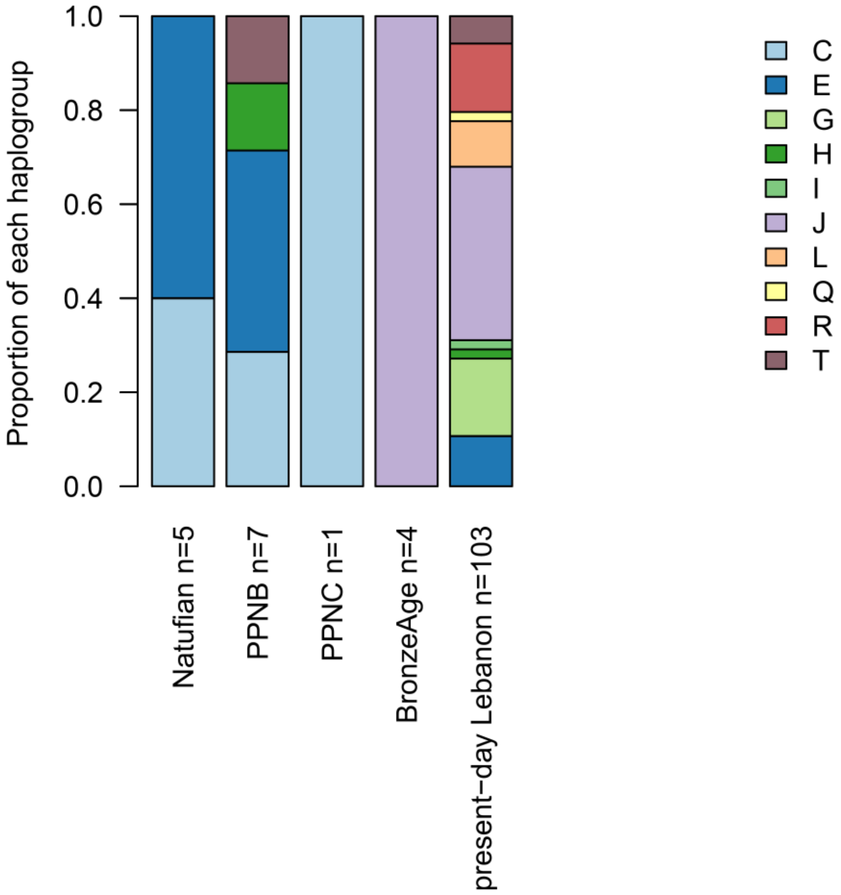

Figure S12. Y-chromosome lineages frequencies in the Levant from Natufians to present-day populations.

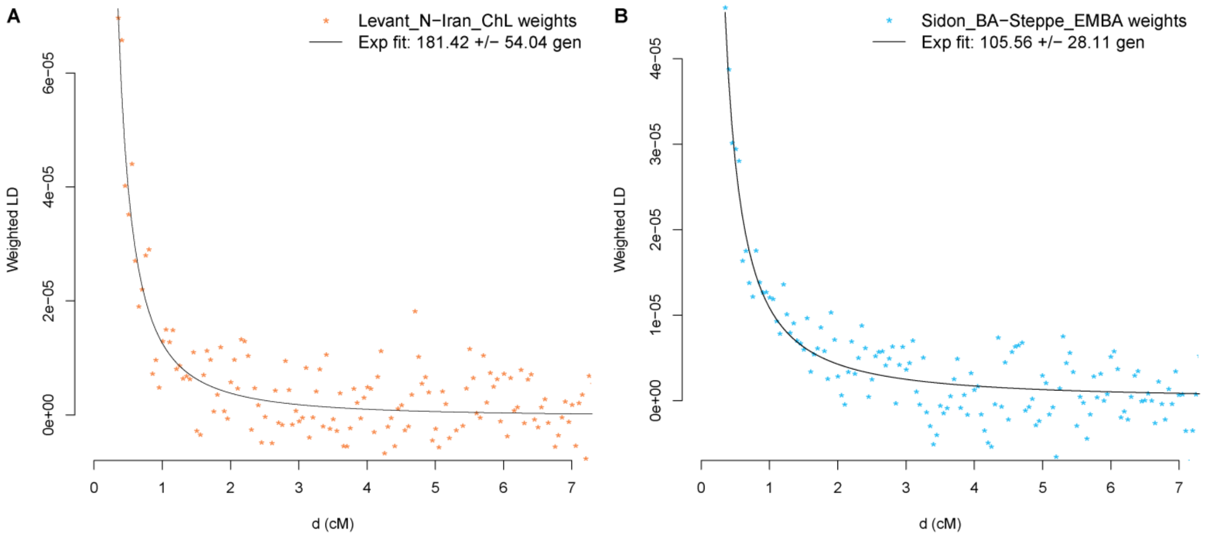

Figure S13. Time of admixture in the Levant. (A) and (B) Weighted LD decay curves for present-day Lebanese using ancient populations as references. (A) Using a generation time of 28 years; a mixture between populations related to Neolithic Levant and Chalcolithic Iran appears to have occurred 6,600-3,550 years ago and (B) mixture between populations related to Bronze Age Sidon and Bronze Age steppe occurred 3,750-2,170 years ago.

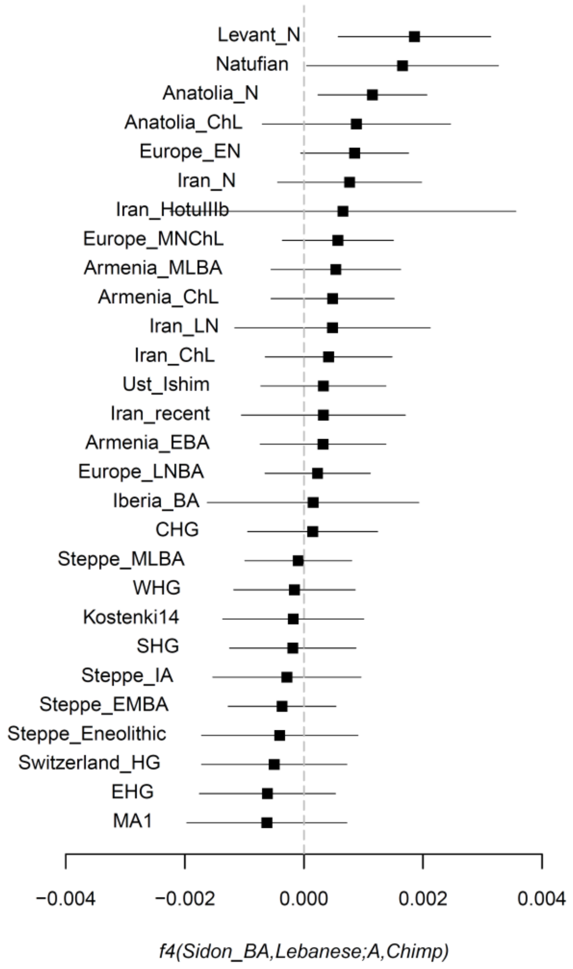

Figure S14. Admixture in present-day Levantine populations (transversions only). We replicate the statistic *f4*(Sidon_BA, Lebanese; Ancient Eurasian, Chimpanzee) using only transversions and get results qualitatively similar to the results from using all variants.

Table S1. Contamination estimates.

Sample	From mtDNA analysis (CI)[a]	From male X chromosome analysis (SE)[b]
**ERS1790732**	0.01 (0-0.02)	
Method1[†]		0.012 (0.002)
Method2		0.014 (0.003)
**ERS1790733**	0.01 (0-0.02)	
Method1		0.017 (0.005)
Method2		0.019 (0.005)
**ERS1790729**	0.01 (0-0.02)	
**ERS1790730**	0.01 (0-0.02)	
**ERS1790731**	0.01 (0-0.02)	

[a]Using schmutzi[3]
[b]Two tests using ANGSD based on methods described in Rasmussen et al. 2011 [4]

Table S2. Phenotypic and disease traits in Sidon_BA. List of putatively functional variants previously identified in the Levant region and their allele frequencies in present-day populations as well as Bronze Age Sidon. We include the read counts as guidance to allele authenticity: for example little supported C>T and G>A variations should be taken with caution.

							AF in present-day populations				AF in Bronze Age Sidon	Read counts in ancient samples				Sidon samples with coverage
Chr	Position	rs ID	Ref	Alt	Gene	Phenotype	AF_Lebanese	AF_AFR	AF_ASN	AF_EUR	AF_Sidon	total A	total C	total G	total T	n
2	136608646	rs4988235	G	A	MCM6	Lactose tolerance	0.02	0.03	0	0.51	0.00	0	0	6	0	3
5	33951693	rs16891982	C	G	SLC45A2	Pigmentation	0.64	0.04	0.01	0.94	0.00	0	9	0	0	4
5	33963870	rs26722	C	T	SLC45A2	Pigmentation	0.11	0.04	0.39	0.02	0.49	0	7	0	5	4
15	28365618	rs12913832	A	G	HERC2	Pigmentation	0.27	0.03	0	0.64	0.27	14	0	3	0	4
15	48426484	rs1426654	A	G	SLC24A5	Pigmentation	0.10	0.93	0.99	0	0.00	16	0	0	0	4
4	72618334	rs7041	A	C	GC	Vitamin D levels	0.55	0.09	0.3	0.58	0.58	3	7	0	0	5
11	71152258	rs736894	C	T	DHCR7	Vitamin D levels	0.34	0.49	0.41	0.26	0.50	0	6	0	4	4
7	141672604	rs10246939	T	C	TAS2R38	Bitter taste perception	0.54	0.48	0.68	0.46	0.36	0	5	0	10	5
9	94118258	rs10991898	C	T	AUH	3-Methylglutaconic aciduria type I	0.20	0.02	0.17	0.11	0.19	0	5	0	2	4
4	100504566	rs61733139	G	C	MTTP	Abetalipoproteinemia	0.01	0.05	0	0.05	0.11	1	0	8	0	5
4	100504664	rs3816873	T	C	MTTP	Abetalipoproteinemia	0.33	0.26	0.14	0.26	0.11	0	1	0	10	4
4	100516022	rs2306985	C	G	MTTP	Abetalipoproteinemia	0.47	0.82	0.67	0.32	0.45	0	3	0	1	3
4	1803704	rs2234909	T	C	FGFR3	Achondroplasia	0.17	0.37	0.07	0.17	0.21	0	1	0	5	4
11	5247141	rs1609812	G	A	HBB	Beta thalassemia major	0.87	0.88	0.51	0.83	0.90	10	0	2	0	5
11	5248050	rs35004220	C	T	HBB	Beta thalassemia major	0.05	0	0	0	0.08	0	10	0	1	4
15	91326099	rs2227935	C	T	BLM	Bloom syndrome	0.03	0.07	0	0.07	0.20	0	8	0	2	5
17	41244936	rs799917	G	A	BRCA1	Breast-ovarian cancer	0.44	0.89	0.37	0.36	0.22	4	0	11	0	5
6	12903957	rs9349379	A	G	PHACTR1	CAD	0.35	0.03	0.69	0.4	0.33	3	0	2	0	4
9	22098574	rs4977574	A	G	CDKN2B-AS1	CAD	0.62	0.14	0.53	0.49	0.35	5	0	3	0	4
9	107620867	rs2230806	C	T	ABCA1	CAD in familial hypercholesterolemia, protection against	0.43	0.71	0.41	0.24	0.70	0	5	0	11	5
1	53712727	rs5174	C	T	LRP8	Myocardial infarction	0.21	0.02	0.03	0.4	0.00	0	6	0	0	3
6	31540313	rs909253	A	G	LTA	Myocardial infarction	0.23	0.52	0.47	0.31	0.00	11	0	0	0	4
12	10313448	rs11053646	C	G	OLR1	Myocardial infarction	0.06	0.22	0.17	0.08	0.21	0	9	2	1	5
14	35761675	rs1048990	C	G	PSMA6	Myocardial infarction	0.22	0.03	0.37	0.16	0.00	0	6	0	0	3
17	45368337	rs15908	A	C	ITGB3	Myocardial infarction	0.48	0.41	0.55	0.37	0.73	3	8	0	0	4
13	113772707	rs6041	G	A	F7	Myocardial infarction, decreasedsusceptibility to	0.23	0.12	0.05	0.11	0.29	2	0	4	0	4
6	6174866	rs5982	G	A	F13A1	Myocardial infarction, protection against	0.27	0.15	0.36	0.21	0.44	3	0	4	0	3
12	121416650	rs1169288	A	C	HNF1A	Diabetes mellitus, noninsulin-dependent, 2	0.49	0.08	0.39	0.34	0.35	3	2	0	0	3
12	121416864	rs1800574	C	T	HNF1A	Diabetes mellitus, noninsulin-dependent, 2	0.05	0	0	0.03	0.16	0	8	0	1	5
4	6302519	rs1801212	G	A	WFS1	Diabetes mellitus, noninsulin-dependent, association with	0.80	0.99	1	0.73	1.00	14	0	0	0	4
4	6302889	rs1801208	G	A	WFS1	Diabetes mellitus, noninsulin-dependent, association with	0.07	0.03	0.08	0.05	0.22	1	0	5	0	4
7	44189393	rs144723656	G	A	GCK	Diabetes mellitus, noninsulin-dependent, late onset	0.00	0	0	0.01	0.00	0	0	12	0	4
8	118184783	rs13266634	C	T	SLC30A8	Diabetes mellitus, noninsulin-dependent, susceptibility to	0.25	0.07	0.46	0.28	0.25	0	13	0	3	4
2	182542998	rs8192556	G	T	NEUROD1	T2D	0.05	0	0	0.02	0.11	1	0	17	0	4
2	227660544	rs1801278	C	T	IRS1	T2D	0.04	0.06	0.02	0.09	0.14	0	4	0	1	3
3	185511687	rs4402960	G	T	IGF2BP2	T2D	0.32	0.57	0.25	0.3	0.33	1	0	3	3	3
3	185529080	rs1470579	A	C	IGF2BP2	T2D	0.33	0.85	0.26	0.3	0.51	6	6	0	0	5
6	20679709	rs7756992	A	G	CDKAL1	T2D	0.29	0.63	0.47	0.28	0.07	13	0	1	0	4
7	127251188	rs712701	T	G	PAX4	T2D	0.79	0.73	0.36	0.78	0.67	1	0	5	2	4
10	114808902	rs12255372	G	T	TCF7L2	T2D	0.32	0.3	0.01	0.29	0.61	0	0	6	9	4
11	2857194	rs2237895	A	C	KCNQ1	T2D	0.38	0.15	0.33	0.42	0.35	3	2	0	0	2
11	17408630	rs5215	C	T	KCNJ11	T2D	0.71	0.95	0.66	0.65	0.50	0	3	0	2	4
11	17409069	rs5218	G	A	KCNJ11	T2D	0.14	0.03	0.51	0.32	0.15	2	0	9	0	4
11	44280090	rs729287	C	T	ALX4	T2D	0.21	0.06	0.45	0.27	0.22	0	10	0	4	4
17	36047417	rs3110641	G	A	HNF1B	T2D	0.32	0.59	0.23	0.26	0.55	3	0	5	0	4
17	36098040	rs4430796	A	G	HNF1B	T2D	0.55	0.69	0.28	0.48	0.45	9	0	7	0	4
20	43058267	rs147638455	A	G	HNF4A	T2D	0.01	0	0	0	0.00	7	0	0	0	4
3	10328453	rs4684677	T	A	GHRL	Obesity	0.03	0.01	0.01	0.07	0.00	0	0	0	5	3
5	148206440	rs1042713	G	A	ADRB2	Obesity	0.47	0.52	0.55	0.39	0.34	2	0	5	0	4
5	148206885	rs1800888	C	T	ADRB2	Obesity	0.02	0	0	0.02	0.00	0	7	0	0	3
5	149212243	rs7732671	G	C	PPARGC1B	Obesity	0.13	0.18	0.06	0.08	0.00	0	0	5	0	3
6	132212694	rs7754561	A	G	ENPP1	Obesity	0.52	0.87	0.55	0.27	0.55	2	0	4	0	3
8	37823798	rs4994	A	G	ADRB3	Obesity	0.07	0.9	0.13	0.8	0.00	12	0	0	0	4
16	53800954	rs1421085	T	C	FTO	Obesity	0.48	0.06	0.17	0.43	0.00	0	0	0	4	3
18	58039276	rs2229616	C	T	MC4R	Obesity, autosomal dominant	0.00	0.02	0.02	0.02	0.00	0	7	0	0	5
16	67516945	rs5030980	C	T	AGRP	Obesity, late-onset	0.08	0.01	0	0.03	0.00	0	10	0	0	5
3	12475557	rs3856806	C	T	PPARG	Obesity, severe	0.11	0.05	0.21	0.12	0.07	0	8	0	0	5
17	3397702	rs12948217	C	T	ASPA	Canavan disease	0.29	0.23	0.05	0.31	0.55	0	3	0	2	4
5	131676320	rs1050152	C	T	SLC22A4	Celiac disease,Crohn's disease	0.33	0.02	0	0.39	0.34	0	5	0	3	3
14	81575005	rs3783941	C	A	TSHR	Congenital Hypo Thyroidsm	0.76	0.64	0.39	0.67	0.75	7	2	0	0	4
7	117199533	rs213950	G	A	CFTR	Cystic fibrosis	0.28	0.93	0.39	0.44	0.21	3	0	6	0	3
9	111651620	rs3204145	A	T	IKBKAP	Dysautonomia, familial	0.17	0.21	0.29	0.17	0.23	4	0	0	1	2
1	169511755	rs4524	T	C	F5	Factor V	0.26	0.17	0.25	0.25	0.52	0	4	0	4	4
1	169519049	rs6025	C	T	F5	Factor V	0.08	0	0	0.01	0.00	0	5	0	0	2
16	3292874	rs2741918	C	T	MEFV	Familial Mediterranean fever	0.57	0.67	0.61	0.56	0.63	0	3	0	5	4
16	3304463	rs224222	C	T	MEFV	Familial Mediterranean fever	0.20	0.04	0.03	0.28	0.08	0	13	0	2	4
9	97873957	rs4647534	A	G	FANCC	Fanconi anemia, complementation groupC	0.39	0.6	0.63	0.45	0.40	6	0	2	0	4
1	100316589	rs2307130	A	G	AGL	Glycogen storage disease IIIb	0.40	0.22	0.54	0.46	0.25	8	0	3	0	5
8	19819724	rs328	C	G	LPL	HDL cholesterol	0.08	0.06	0.12	0.13	0.29	0	10	6	0	5
8	27373865	rs751141	G	A	EPHX2	Hypercholesterolemia, familial, due to LDLR defect, modifier of	0.05	0.11	0.23	0.09	0.00	0	0	8	0	4
5	42700044	rs6179	A	G	GHR	Hypercholesterolemia, familial, modifier	0.67	0.4	0.82	0.68	0.21	6	0	1	0	4
5	42719239	rs6180	A	C	GHR	Hypercholesterolemia, familial, modifier	0.47	0.38	0.57	0.42	0.26	2	1	0	1	3
11	17417496	rs1800853	G	A	ABCC8	Hyperinsulinemic hypoglycemia, familial, 1	0.02	0.04	0.03	0.01	0.10	1	0	13	0	5
7	113518434	rs1799999	C	A	PPP1R3A	Insulin resistance, severe, digenic	0.16	0.18	0.64	0.12	0.00	0	6	0	0	5
12	112939853	rs3741983	C	T	PTPN11	Insulin resistance, susceptibility to	0.27	0.8	0.84	0.27	0.70	0	5	0	5	4
19	11241915	rs6413504	A	G	LDLR	LDL cholesterol	0.50	0.14	0.3	0.48	0.36	10	0	6	0	5
4	38799710	rs4833095	T	C	TLR1	Leprosy, immunity	0.49	0.88	0.6	0.28	0.15	0	1	0	10	5
1	100672060	rs12021720	T	C	DBT	Maple syrup urine disease, type II	0.89	0.74	0.95	0.92	1.00	0	10	0	0	4
15	72643444	rs117160567	A	C	HEXA	Tay-Sachs disease	0.01	0	0	0.02	0.00	15	0	0	0	4
21	44480616	rs1801181	G	A	CBS	Thrombosis, hyperhomocysteinemic	0.34	0.02	0.57	0.39	0.28	3	0	4	0	3
10	56129066	rs11594958	A	G	PCDH15	Usher syndrome, type 1F	0.25	0.22	0.09	0.22	0.18	6	0	3	0	4
1	68903942	rs12145904	C	T	RPE65	Vitamin A metabolic defect	0.11	0.23	0.36	0.13	0.14	0	5	0	1	2
9	134385599	rs2018621	A	G	POMT1	Walker–Warburg syndrome	0.94	0.72	0.92	0.94	0.83	1	0	8	0	5
14	77746310	rs438931	G	A	POMT2	Walker–Warburg syndrome	0.09	0.13	0.33	0.13	0.00	0	0	10	0	4

Table S3. Modeling Sidon_BA as Levant_N and an ancient population *A*. Sidon_BA can be modelled as a mix of Neolithic Levant 0.484±0.042 and Chalcolithic Iran 0.516±0.042 using a large number of outgroups: Ust_Ishim, Kostenki14, MA1, Han, Papuan, Ami, Chukchi, Karitiana, Mbuti, Switzerland_HG, EHG, WHG, and CHG.

		Mixture Proportions		
**A**	**P-value for rank=1**	**Levant_N**	**A**	**Std. Error**
Iran_ChL	27.89E-02	0.484	0.516	0.042
Iran_HotuIIIb	2.50E-02	0.593	0.407	0.048
Iran_LN	2.45E-02	0.559	0.441	0.044
Iran_recent	1.16E-02	0.309	0.691	0.089
Natufian	0.94E-02	2.33	-1.33	0.376
Iran_N	0.45E-02	0.592	0.408	0.041
Armenia_EBA	0.02E-02	0.528	0.472	0.046
Anatolia_ChL	2.57E-07	0.324	0.676	0.123
Armenia_MLBA	8.78E-11	0.653	0.347	0.052
Europe_MNChL	6.11E-13	1.261	-0.261	0.053
Iberia_BA	3.82E-13	1.375	-0.375	0.107
Armenia_ChL	4.97E-14	0.692	0.308	0.063
Europe_EN	5.73E-15	1.475	-0.475	0.142
SHG	3.50E-16	1.072	-0.072	0.017
Anatolia_N	6.71E-17	1.746	-0.746	0.411
Steppe_IA	4.02E-18	0.891	0.109	0.039
Steppe_EMBA	7.68E-19	0.928	0.072	0.027
Europe_LNBA	6.90E-20	1.053	-0.053	0.04
Steppe_Eneolithic	5.79E-20	0.978	0.022	0.018
Steppe_MLBA	2.71E-20	1.005	-0.005	0.036

Table S4. Counts of Y-chromosome ancestral and derived allele genotypes in the Sidon_BA males

	ERS1790733 observed genotypes			ERS1790732 observed genotypes	
Haplogroup	Number of ancestral alleles	Number of derived alleles	Haplogroup	Number of ancestral alleles	Number of derived alleles
A00	22	0	A00	26	0
A0-T	0	7	A0-T	0	15
A0	17	0	A0	12	0
A1	0	4	A1	0	4
A1a	8	0	A1a	7	0
A1b	1	14	A1b	1	15
BT	0	121	BT	0	171
B	1	0	B	1	0
CT	0	73	CT	1	106
DE	10	0	DE	13	0
CF	0	2	CF	0	1
C	2	0	C	5	0
F	0	10	F	0	17
F1	2	0	F1	2	0
F3	1	0	F3	1	0
GHIJK	0	1	GHIJK	0	1
G	70	1	G	81	1
H	8	0	H	10	0
IJK	0	2	IJK	0	2
IJ	0	2	IJ	0	3
I	51	1	I	63	0
J	0	31	J	0	40
J1	0	1	J1	1	0
J2	1	0	J2	0	1
J1a	0	1	J1a	1	0
J2a	2	0	J2a	3	0
J2b	2	0	J2b	0	2
J1a3	1	0	J2b2a	1	0
J1a2a	1	0	J2b2a1	1	0
J1a2b	0	1	J2b2a1a	1	0
J1a2b3	1	0	J2b2a1a1	1	0
J1a2b3a	1	0			

Table S5. mtDNA haplogroups frequencies in present-day populations and Sidon_BA

Population (n)	A	B	C	D	F	G	H	HV	I	J1	J2	K	L0	L1	L2	L3	L4	L5	L6	M	N	R	T1	T2	U1	U2	U3	U4	U5	U6	U7	U8	V	W	X	Z3
**Egyptian (100)**							0.20	0.04	0.02	0.03	0.02	0.04		0.04	0.06	0.10	0.01			0.03	0.07	0.04	0.08	0.09	0.01	0.01	0.01		0.01	0.02	0.01	0.01			0.05	
**Ethiopian (124)**								0.03	0.01	0.02		0.02	0.09	0.02	0.13	0.32	0.06	0.04	0.02	0.09	0.06	0.08				0.01		0.01								
**Greek (100)**			0.01				0.44			0.07	0.01	0.02								0.01	0.01	0.04	0.03	0.07	0.02	0.01	0.01	0.01	0.13		0.02	0.01	0.03	0.02	0.03	
**Lebanese (103)**							0.20	0.09	0.03	0.08	0.03	0.08	0.01							0.01	0.08	0.11	0.05	0.06	0.06	0.01	0.03	0.01			0.02	0.03		0.02	0.01	
**CEU (99)**							0.51	0.01	0.01	0.06	0.02	0.03											0.03	0.07		0.02	0.01	0.02	0.13				0.03	0.05		
**Han (103)**	0.07	0.09	0.05	0.22	0.16	0.05						0.01								0.18	0.08	0.09														0.01
**YRI (109)**													0.05	0.16	0.35	0.44	0.01																			
**Sidon_BA (5)**							0.20	0.20				0.20									0.20	0.20														

Table S6: Modelling Lebanese as Sidon_BA and an ancient population *A*. Lebanese can be modelled as a mix of Bronze Age Sidon 0.93±0.018 and a steppe ancestry 0.07±0.018 using outgroups: Ust_Ishim, Kostenki14, MA1, Han, Papuan, Ami, Chukchi, Karitiana, Mbuti, Switzerland_HG, EHG, WHG, and CHG.

		Mixture Proportions		
**A**	**P-value for rank=1**	**Sidon_BA**	**A**	**Std. Error**
Steppe_MLBA	9.49E-02	0.924	0.076	0.018
Steppe_EMBA	9.48E-02	0.932	0.068	0.016
Steppe_IA	9.28E-02	0.92	0.08	0.02
Europe_LNBA	5.14E-02	0.927	0.073	0.019
Steppe_Eneolithic	5.14E-02	0.962	0.038	0.011
Iran_LN	3.19E-02	1.16	-0.16	0.063
SHG	3.01E-02	0.972	0.028	0.008
Iberia_BA	1.54E-02	0.934	0.066	0.022
Iran_recent	1.24E-02	0.69	0.31	0.151
Armenia_MLBA	1.04E-02	0.882	0.118	0.041
Armenia_ChL	7.52E-03	0.887	0.113	0.042
Iran_N	5.70E-03	1.104	-0.104	0.055
Natufian	3.93E-03	1.08	-0.08	0.042
Europe_MNChL	2.69E-03	0.962	0.038	0.019
Anatolia_ChL	2.10E-03	0.881	0.119	0.087
Mota	1.64E-03	1.007	-0.007	0.004
Europe_EN	1.51E-03	0.95	0.05	0.033
Iran_ChL	1.15E-03	1.087	-0.087	0.094
Anatolia_N	7.98E-04	0.968	0.032	0.049
Iran_HotuIIIb	7.34E-04	1.023	-0.023	0.068
Armenia_EBA	6.77E-04	0.989	0.011	0.068

Supplemental references

1. Lazaridis, I., Nadel, D., Rollefson, G., Merrett, D.C., Rohland, N., Mallick, S., Fernandes, D., Novak, M., Gamarra, B., Sirak, K., et al. (2016). Genomic insights into the origin of farming in the ancient Near East. Nature 536, 419-424.
2. Haber, M., Gauguier, D., Youhanna, S., Patterson, N., Moorjani, P., Botigue, L.R., Platt, D.E., Matisoo-Smith, E., Soria-Hernanz, D.F., Wells, R.S., et al. (2013). Genome-wide diversity in the levant reveals recent structuring by culture. PLoS genetics 9, e1003316.
3. Renaud, G., Slon, V., Duggan, A.T., and Kelso, J. (2015). Schmutzi: estimation of contamination and endogenous mitochondrial consensus calling for ancient DNA. Genome biology 16, 224.
4. Rasmussen, M., Guo, X., Wang, Y., Lohmueller, K.E., Rasmussen, S., Albrechtsen, A., Skotte, L., Lindgreen, S., Metspalu, M., Jombart, T., et al. (2011). An Aboriginal Australian genome reveals separate human dispersals into Asia. Science 334, 94-98.
