# Supplementary material for: Continuity and Admixture in the Last Five Millennia of Levantine History from Ancient Canaanite and Present-Day Lebanese Genome Sequences
Source: Am J Hum Genet. 2017 Jul 27;101(2):274–82. doi: 10.1016/j.ajhg.2017.06.013 (PMC5544389; doi:10.1016/j.ajhg.2017.06.013)
Supplement: Document S2. Article plus Supplemental Data [file mmc2.pdf]

REPORT

Continuity and Admixture in the Last Five Millennia of Levantine History from Ancient Canaanite and Present-Day Lebanese Genome Sequences

Marc Haber,[1,9,*] Claude Doumet-Serhal,[2,9] Christiana Scheib,[3,9] Yali Xue,[1] Petr Danecek,[1] Massimo Mezzavilla,[1] Sonia Youhanna,[4] Rui Martiniano,[1] Javier Prado-Martinez,[1] Michał Szpak,[1] Elizabeth Matisoo-Smith,[5] Holger Schutkowski,[6] Richard Mikulski,[6] Pierre Zalloua,[7,8] Toomas Kivisild,[3] and Chris Tyler-Smith[1,*]

The Canaanites inhabited the Levant region during the Bronze Age and established a culture that became influential in the Near East and beyond. However, the Canaanites, unlike most other ancient Near Easterners of this period, left few surviving textual records and thus their origin and relationship to ancient and present-day populations remain unclear. In this study, we sequenced five whole genomes from ~3,700-year-old individuals from the city of Sidon, a major Canaanite city-state on the Eastern Mediterranean coast. We also sequenced the genomes of 99 individuals from present-day Lebanon to catalog modern Levantine genetic diversity. We find that a Bronze Age Canaanite-related ancestry was widespread in the region, shared among urban populations inhabiting the coast (Sidon) and inland populations (Jordan) who likely lived in farming societies or were pastoral nomads. This Canaanite-related ancestry derived from mixture between local Neolithic populations and eastern migrants genetically related to Chalcolithic Iranians. We estimate, using linkage-disequilibrium decay patterns, that admixture occurred 6,600–3,550 years ago, coinciding with recorded massive population movements in Mesopotamia during the mid-Holocene. We show that present-day Lebanese derive most of their ancestry from a Canaanite-related population, which therefore implies substantial genetic continuity in the Levant since at least the Bronze Age. In addition, we find Eurasian ancestry in the Lebanese not present in Bronze Age or earlier Levantines. We estimate that this Eurasian ancestry arrived in the Levant around 3,750–2,170 years ago during a period of successive conquests by distant populations.

The Near East, including the Levant, has been central to human prehistory and history from the expansion out of Africa 50–60 thousand years ago (kya),[1] through post-glacial expansions[2] and the Neolithic transition 10 kya, to the historical period when Ancient Egyptians, Greeks, Phoenicians, Assyrians, Babylonians, Persians, Romans, and many others left their impact on the region.[3] Aspects of the genetic history of the Levant have been inferred from present-day DNA,[4,5] but the more comprehensive analyses performed in Europe[6–11] have shown the limitations of relying on present-day information alone and highlighted the power of ancient DNA (aDNA) for addressing questions about population histories.[12] Unfortunately, although the few aDNA results from the Levant available so far are sufficient to reveal how much its history differs from that of Europe,[13] more work is needed to establish a thorough understanding of Levantine genetic history. Such work is hindered by the hot and sometimes wet environment,[12,13] but improved aDNA technologies including use of the petrous bone as a source of DNA[14] and the rich archaeological remains available encouraged us to further explore the potential of aDNA in this region. Here, we present genome sequences from five Bronze Age Lebanese samples and show how they improve our understanding of the Levant's history over the last five millennia.

During the Bronze Age in the Levant, around 3–4 kya, a distinctive culture emerged as a Semitic-speaking people known as the Canaanites. The Canaanites inhabited an area bounded by Anatolia to the north, Mesopotamia to the east, and Egypt to the south, with access to Cyprus and the Aegean through the Mediterranean. Thus the Canaanites were at the center of emerging Bronze Age civilizations and became politically and culturally influential.[15] They were later known to the ancient Greeks as the Phoenicians who, 2.3–3.5 kya, colonized territories throughout the Mediterranean reaching as far as the Iberian Peninsula.[16] However, for uncertain reasons but perhaps related to the use of papyrus instead of clay for documentation, few textual records have survived from the Canaanites themselves and most of their history known today has been reconstructed from ancient Egyptian and Greek records, the Hebrew Bible, and archaeological excavations.[15] Many uncertainties still surround the origin of the Canaanites. Ancient Greek historians believed their homeland was located in the region of the Persian Gulf,[16,17] but modern researchers tend to reject this hypothesis because of archaeological and historical evidence

[1]Wellcome Trust Sanger Institute, Wellcome Genome Campus, Hinxton, Cambridgeshire CB10 1SA, UK; [2]The Sidon excavation, Saida, Lebanon; [3]Department of Archaeology and Anthropology, University of Cambridge, Cambridge CB2 1QH, UK; [4]Institute of Physiology, University of Zurich, Winterthurerstrasse 190, 8057 Zürich, Switzerland; [5]Department of Anatomy, University of Otago, Dunedin 9054, New Zealand; [6]Department of Archaeology, Anthropology, and Forensic Science, Bournemouth University, Talbot Campus, Poole BH12 5BB, UK; [7]The Lebanese American University, Chouran, Beirut 1102 2801, Lebanon; [8]Harvard T.H. Chan School of Public Health, Boston, MA 02115, USA
[9]These authors contributed equally to this work
*Correspondence: mh25@sanger.ac.uk (M.H.), cts@sanger.ac.uk (C.T.-S.)
http://dx.doi.org/10.1016/j.ajhg.2017.06.013.
© 2017 The Author(s). This is an open access article under the CC BY license (http://creativecommons.org/licenses/by/4.0/).

Table 1. Samples Analyzed in This Study

ENA Number	Burial Number	Time Years Ago	Mapped Reads[a]	Mapped Read %	Coverage Genomic	Coverage MT	Sex[b]	MT Haplogroup	Y Haplogroup
ERS1790733	54	3,700[c]	69,084,826	6.24	1.19	110	M	N1a3a	J1-P58
ERS1790732	63	3,650[d]	98,293,308	9.20	1.69	109	M	HV1b1	J2-M12
ERS1790730	65	3,650[d]	73,701,096	7.57	1.24	124	F	K1a2	–
ERS1790731	75	3,750[d]	128,355,897	15.48	2.32	164	F	R2	–
ERS1790729	46	3,750[d]	23,323,399	2.64	0.40	53	F	H1bc	–

[a]Excluding PCR duplicates
[b]Genetically determined
[c]Radiocarbon date
[d]Archaeological date

of population continuity through successive millennia in the Levant. The Canaanite culture is alternatively thought to have developed from local Chalcolithic people who were themselves derived from people who settled in farming villages 9–10 kya during the Neolithic period.[15] Uncertainties also surround the fate of the Canaanites: the Bible reports the destruction of the Canaanite cities and the annihilation of its people; if true, the Canaanites could not have directly contributed genetically to present-day populations. However, no archaeological evidence has so far been found to support widespread destruction of Canaanite cities between the Bronze and Iron Ages: cities on the Levant coast such as Sidon and Tyre show continuity of occupation until the present day.

aDNA research has the potential to resolve many questions related to the history of the Canaanites, including their place of origin and fate. Here, we sampled the petrous portion of temporal bones belonging to five ancient individuals dated to between 3,750 and 3,650 years ago (ya) from Sidon, which was a major Canaanite city-state during this period (Figures S1 and S2). We extracted DNA and built double-stranded libraries according to published protocols without uracil-DNA glycosylase treatment.[18–21] We sequenced the libraries on an Illumina HiSeq 2500 using 2× 75 bp reads and processed the sequences using the PALEOMIX pipeline.[22] We retained reads ≥30 bp and collapsed pairs with minimum overlap of 15 bp, allowing a mismatch rate of 0.06 between the pairs. We mapped the merged sequences to the *hs37d5* reference sequence, removed duplicates, removed two bases from the ends of each read, and randomly sampled a single sequence with a minimum quality of ≥20 to represent each SNP. We obtained a genomic coverage of 0.4–2.3× and a mitochondrial DNA (mtDNA) genome coverage of 53–164× (Table 1). Y chromosome genotypes were jointly called across males from the 1000 Genomes Project, present-day Lebanese, and two identified Canaanite males using FreeBayes v.0.9.18.[23] A maximum likelihood phylogeny was inferred using RAxML v.8.2.10[24] and visualized using iTOL v.3.5.3.[25] In order to assess ancient DNA authenticity, we estimated mtDNA and X chromosome contamination[26–28] (Table S1) and restricted some analyses to sequences with aDNA damage patterns[29,30] (Figures S3 and S4), demonstrating that the sequence data we present are endogenous and minimally contaminated.

Additionally, we sequenced whole genomes of 99 present-day Lebanese individuals with informed consent to ~8× coverage on an Illumina HiSeq 2500 using 2× 100 bp reads in a study approved by The Wellcome Trust Sanger Institute's Human Materials and Data Management Committee (13/010 and 14/072). We merged the low-coverage Lebanese data with four high-coverage (30×) Lebanese samples,[31] 1000 Genomes Project phase 3 CEU, YRI, and CHB populations,[32] and sequence data previously published from regional populations (Egyptians, Ethiopians, and Greeks).[1,31] Raw calls were generated using bcftools (bcftools mpileup -C50 -pm3 -F0.2 -d10000 | bcftools call -mv, version 1.2-239-g8749475) and filtered to include only SNPs with the minimum of two alternate alleles in at least one population and site quality larger than ten; we excluded sites with a minimum per-population HWE and total HWE less than 0.01[33] and sites within 3 bp of an indel. The filtered calls were then pre-phased using shapeit (v.2.r790)[34] and their genotypes refined using beagle (v.4.1).[35] We have previously described the genetic structure in the Lebanese population using array data from ~1,300 individuals.[4] A principal component analysis (PCA) using the 99 sequenced present-day individuals show that they capture the previously described genetic diversity with distinct clusters reflecting the different cultural groups in Lebanon today (Figure S5).

We combined our ancient and modern samples with previously published ancient data[6–11,13,36–38] (Figure 1A) resulting in a dataset of 389 individuals and 1,046,317 SNPs when ancient and Lebanese samples were analyzed, and 546,891 SNPs when 2,583 modern samples from the Human Origins genotype data were included in the analysis (i.e., the small dataset was used only when a modern population other than the Lebanese was included in the test).[9,39] A pooled Lebanese sequence dataset (99 low coverage plus 4 high coverage) was used in all analyses except for the PCA and ADMIXTURE where a subset of 15 randomly selected individuals (5 from each group described in Figure S5) was used to avoid sample size

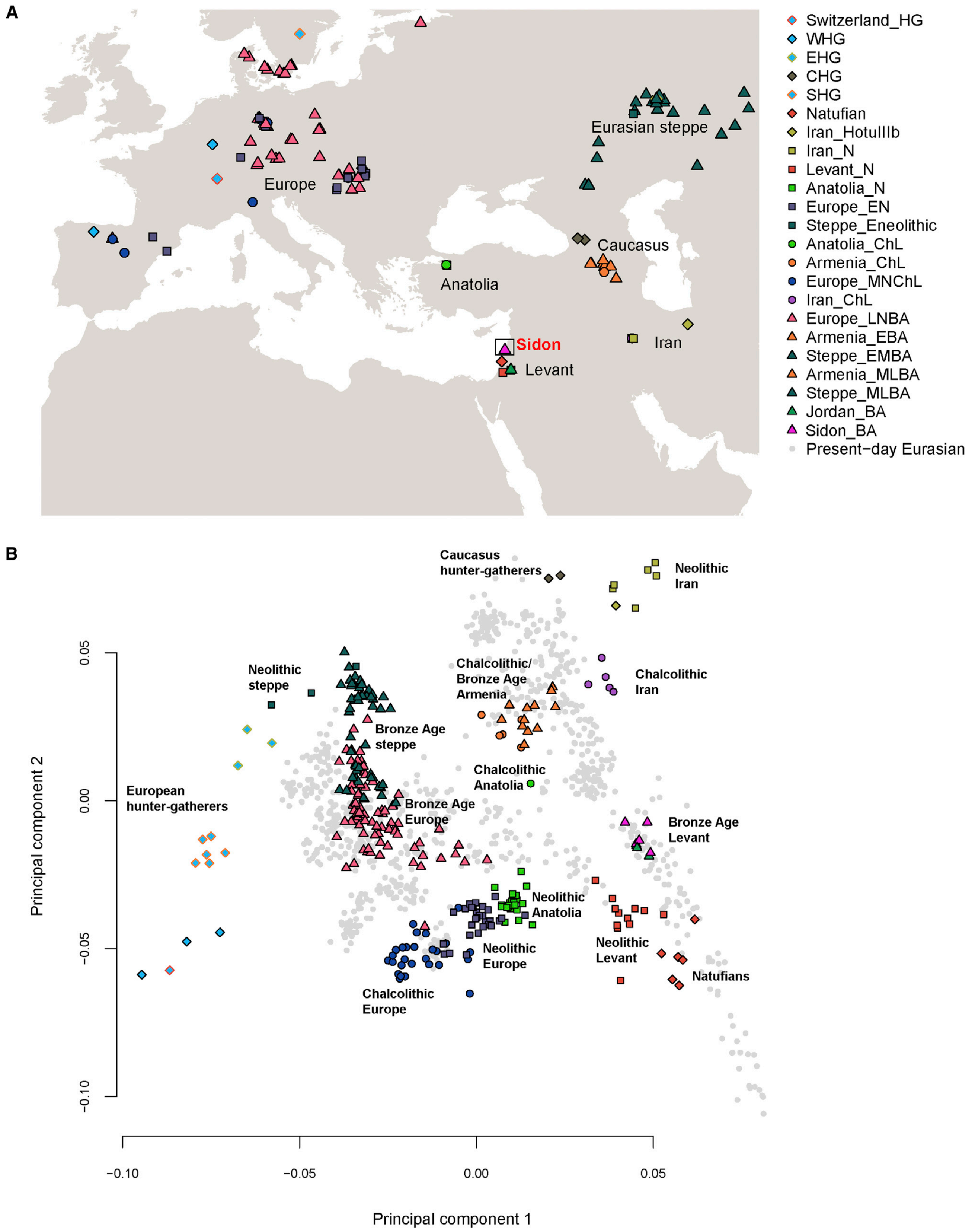

Figure 1. Population Locations and Genetic Structure
(A) The map shows the location of the newly sequenced Bronze Age Sidon samples (pink triangle labeled with red text), as well as the locations of published ancient samples used as comparative data in this study.
(B) PCA of ancient Eurasian samples (colored shapes) projected using eigenvectors from present-day Eurasian populations (gray points).

bias. The ancient samples were grouped following the labels assigned by Lazaridis et al.[13] on the basis of archaeological culture, chronology, and genetic clustering. We used this dataset to shed light on the genetic history of the Canaanites, resolving their relationship to other ancient populations and assessing their genetic contribution to present-day populations.

We first explored our dataset using PCA[40] on present-day West Eurasian (including Levantine) populations and projected the ancient samples onto this plot (Figures 1B and S6). The Bronze Age Sidon samples (Sidon_BA) overlap with present-day Levantines and were positioned between the ancient Levantines (Natufians/Neolithic) and ancient Iranians (Neolithic/Chalcolithic). The overlap between the Bronze Age and present-day Levantines suggests a degree of genetic continuity in the region. We explored this further by computing the statistic *f4*(Lebanese, present-day Near Easterner; Sidon_BA, Chimpanzee) using *qpDstat*[39] (with parameter f4mode: YES) and found that Sidon_BA shared more alleles with the Lebanese than with most other present-day Levantines (Figure S7), supporting local population continuity as observed in Sidon's archaeological records. When we substituted present-day Near Easterners with a panel of 150 present-day populations available in the Human Origins dataset, we found that only Sardinians and Italian_North shared significantly more alleles with Sidon_BA compared with the Lebanese (Figure S8). Sardinians are known to have retained a large proportion of ancestry from Early European farmers (EEFs) and therefore the increased affinity to Sidon_BA could be related to a shared Neolithic ancestry. We computed *f4*(Lebanese, Sardinian/Italian_North; Sidon_BA, Levant_N) and found no evidence of increased affinity of Sardinians or Italian_North to Sidon_BA after the Neolithic (both Z-scores are positive). We next wanted to explore whether the increased affinity of Sidon_BA to the Lebanese could also be observed when analyzing functionally important regions of the genome that are less susceptible to genetic drift. Our sequence data allowed us to scan loci linked to phenotypic traits and loci previously identified as functional variants in the Lebanese and other Levantines.[41–43] Using a list of 84 such variants (Table S2), we estimated the allele frequency (AF) in Sidon_BA using ANGSD[26] based on a method from Li et al.[44] and calculated Pearson pairwise correlation coefficients between AF in Sidon_BA and AF in Africans, Europeans, Asians,[32] and Lebanese. We found a high significant correlation between Sidon_BA and the Lebanese ($r = 0.74$; 95% CI = 0.63–0.82; p value = 8.168×10^{-16}) and lower correlations between Sidon_BA and Europeans ($r = 0.56$), Africans ($r = 0.55$), and Asians ($r = 0.53$) (Figure S9). These results support population continuity in the region and suggest that several present-day genetic disorders might stem from risk alleles that were already present in the Bronze Age population. In addition, SNPs associated with phenotypic traits show that Sidon_BA and the Lebanese had comparable skin, hair, and eye colors (in general: light intermediate skin pigmentation, brown eyes, and dark hair) with similar frequencies of the underlying causal variants in *SLC24A5* and *HERC2*, but with Sidon_BA probably having darker skin than Lebanese today from variants in *SLC45A2* resulting in darker pigmentation (Table S2).

The PCA shows that Sidon_BA clusters with three individuals from Early Bronze Age Jordan (Jordan_BA) found in a cave above the Neolithic site of 'Ain Ghazal and probably associated with an Early Bronze Age village close to the site.[13] This suggests that people from the highly differentiated urban culture on the Levant coast and inland people with different modes of subsistence were nevertheless genetically similar, supporting previous reports that the different cultural groups who inhabited the Levant during the Bronze Age, such as the Ammonites, Moabites, Israelites, and Phoenicians, each achieved their own cultural identities but all shared a common genetic and ethnic root with Canaanites.[15] Lazaridis et al.[13] reported that Jordan_BA can be modeled as mixture of Neolithic Levant (Levant_N) and Chalcolithic Iran (Iran_ChL). We computed the statistic *f4*(Levant_N, Sidon_BA; Ancient Eurasian, Chimpanzee) and found that populations from the Caucasus and ancient Iran shared more alleles with Sidon_BA than with Neolithic Levant (Figure 2A and S10). We then used *qpAdm*[8] (with parameter allsnps: YES) to test whether Sidon_BA can be modeled as mixture of Levant_N and any other ancient population in the dataset and found good support for the model of Sidon_BA being a mixture of Levant_N (48.4% ± 4.2%) and Iran_ChL (51.6% ± 4.2%) (Figure 2B; Table S3).

In addition, the two Sidon_BA males carried the Y-chromosome haplogroups[45] J-P58 (J1a2b) and J-M12 (J2b) (Tables 1 and S4; Figure S11), both common male lineages in the Near East today. Haplogroup J-P58 is frequent in the Arabian peninsula with proposed origins in the Zagros/Taurus mountain region.[46] It forms the vast majority of the Y chromosomes in southwestern Mesopotamia and reaches particularly high frequencies (74.1%) in Marsh Arabs in Iraq.[47] On the other hand, haplogroup J-M12 is widespread at low frequency from the Balkans to India and the Himalayas, with Albanians having the highest proportions (14.3%).[48] We compiled frequencies of Y-chromosome haplogroups in this geographical area and their changes over time in a dataset of ancient and modern Levantine populations (Figure S12), and note, similarly to Lazaridis et al.,[13] that haplogroup J was absent in all Natufian and Neolithic Levant male individuals examined thus far, but emerged during the Bronze Age in Lebanon and Jordan along with ancestry related to Iran_ChL. All five Sidon_BA individuals had different mitochondrial DNA haplotypes[49] (Table 1), belonging to paragroups common in present-day Lebanon and nearby regions (Table S5) but with additional derived variants not observed in our present-day Lebanese dataset.

We next sought to estimate the time when the Iran_ChL-related ancestry penetrated the Levant. Our

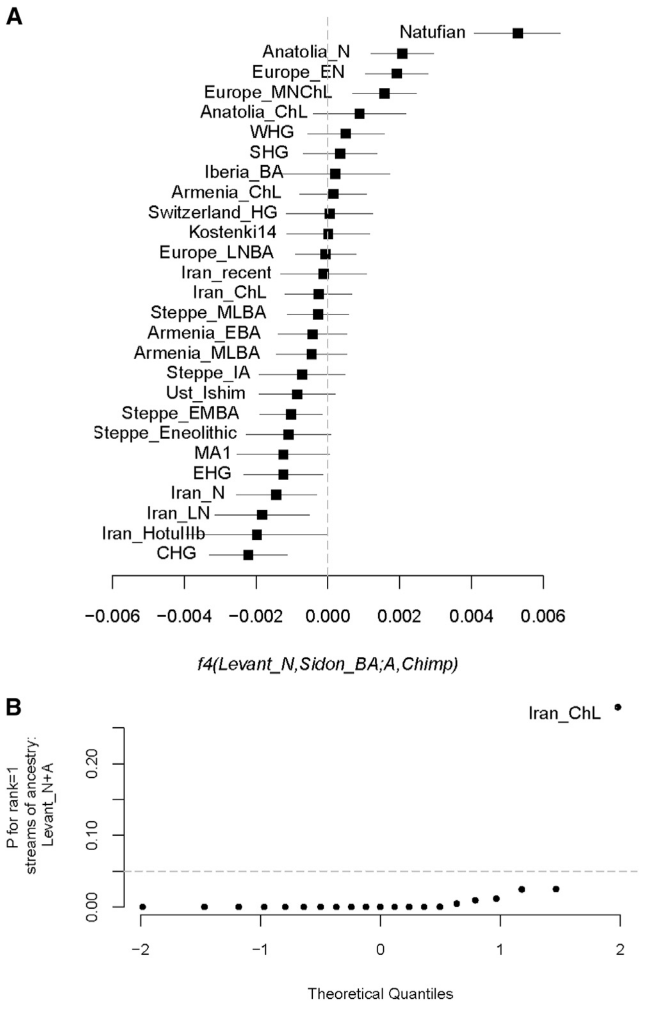

Figure 2. Admixture in Bronze Age Levantine Populations

(A) The statistic *f4*(Levant_N, Sidon_BA; Ancient Eurasian, Chimpanzee) is most negative for ancient populations from the Caucasus and Iran, suggesting an increase in ancestry related to these populations in Sidon after the Neolithic period. The plot shows the estimated statistic value and ± 3 standard errors.

(B) Modeling Sidon as mixture between Neolithic Levant and an ancient Eurasian population shows that Chalcolithic Iran fits the model best when using a large number of outgroups: Ust_Ishim, Kostenki14, MA1, Han, Papuan, Ami, Chukchi, Karitiana, Mbuti, Switzerland_HG, EHG, WHG, and CHG. Sidon_BA can then be modeled using *qpAdm* as 0.484 ± 0.042 Levant_N and 0.516 ± 0.042 Iran_ChL.

results support genetic continuity since the Bronze Age and thus our large dataset of present-day Lebanese provided an opportunity to explore the admixture time using admixture-induced linkage disequilibrium (LD) decay. Using ALDER[50] (with mindis: 0.005), we set the Lebanese as the admixed test population and Natufians, Levant_N, Sidon_BA, Iran_N, and Iran_ChL as reference populations. To account for the small number of individuals in the reference populations and the limited number of SNPs in the dataset, we took a lenient minimum Z-score = 2 to be suggestive of admixture. The most significant result was for mixture of Levant_N and Iran_ChL ($p = 0.013$) around 181 ± 54 generations ago, or ~5,000 ± 1,500 ya assuming a generation time of 28 years (Figure S13A). This admixture time, based entirely on genetic data, fits the known ages of the samples based on archaeological data since it falls between the dates of Sidon_BA (3,650–3,750 ya) and Iran_ChL (6,500–5,500 ya). The admixture time also overlaps with the rise and fall of the Akkadian Empire which controlled the region from Iran to the Levant between ~4.4 and 4.2 kya. The Akkadian collapse is argued to have been the result of a widespread aridification event around 4,200 ya.[51,52] Archaeological evidence in this period documents large-scale influxes of refugees from Northern Mesopotamia toward the south, where cities and villages became overpopulated.[53] Our confidence intervals for the admixture dates are wide and therefore the historical links suggested here should be considered with caution. Future sampling of ancient DNA from northern Syria and Iraq will reveal whether these populations carried the Iran_ChL-related ancestry and also provide a better understanding of the origin of the eastern migrants and the time when they arrived in the Levant.

Although *f4* tests showed that present-day Lebanese share significantly more alleles with Sidon_BA than other Near Eastern populations do, indicating genetic continuity, we failed to model the present-day Lebanese using streams of ancestry coming only from Levant_N and Iran_ChL (*qpAdm* rank1 $p = 8.36 \times 10^{-7}$), in contrast to our success with Sidon_BA. We therefore further explored our dataset by running ADMIXTURE[54] in a supervised mode using Western hunter-gatherers (WHG), Eastern hunter-gatherers (EHG), Levant_N, and Iran_N as reference populations. These four populations have been previously[13] found to contribute genetically to most West Eurasians. The ADMIXTURE results replicate the findings from *qpAdm* for Sidon_BA and show mixture of Levant_N and ancient Iranian populations (Figure 3A). However, the present-day Lebanese, in addition to their Levant_N and ancient Iranian ancestry, have a component (11%–22%) related to EHG and Steppe populations not found in Bronze Age populations (Figure 3A). We confirm the presence of this ancestry in the Lebanese by testing *f4*(Sidon_BA, Lebanese; Ancient Eurasian, Chimpanzee) and find that Eurasian hunter-gatherers and Steppe populations share more alleles with the Lebanese than with Sidon_BA (Figures 3B and S14). We next tested a model of the present-day Lebanese as a mixture of Sidon_BA and any other ancient Eurasian population using *qpAdm*. We found that the Lebanese can be best modeled as Sidon_BA 93% ± 1.6% and a Steppe Bronze Age population 7% ± 1.6% (Figure 3C; Table S6). To estimate the time when the Steppe ancestry penetrated the Levant, we used, as above, LD-based inference and set the Lebanese as admixed test population with Natufians, Levant_N, Sidon_BA, Steppe_EMBA, and Steppe_MLBA as reference populations. We found support ($p = 0.00017$) for a mixture between Sidon_BA and Steppe_EMBA which has occurred around 2,950 ± 790 ya (Figure S13B). It is important to note here that Bronze Age Steppe populations used in

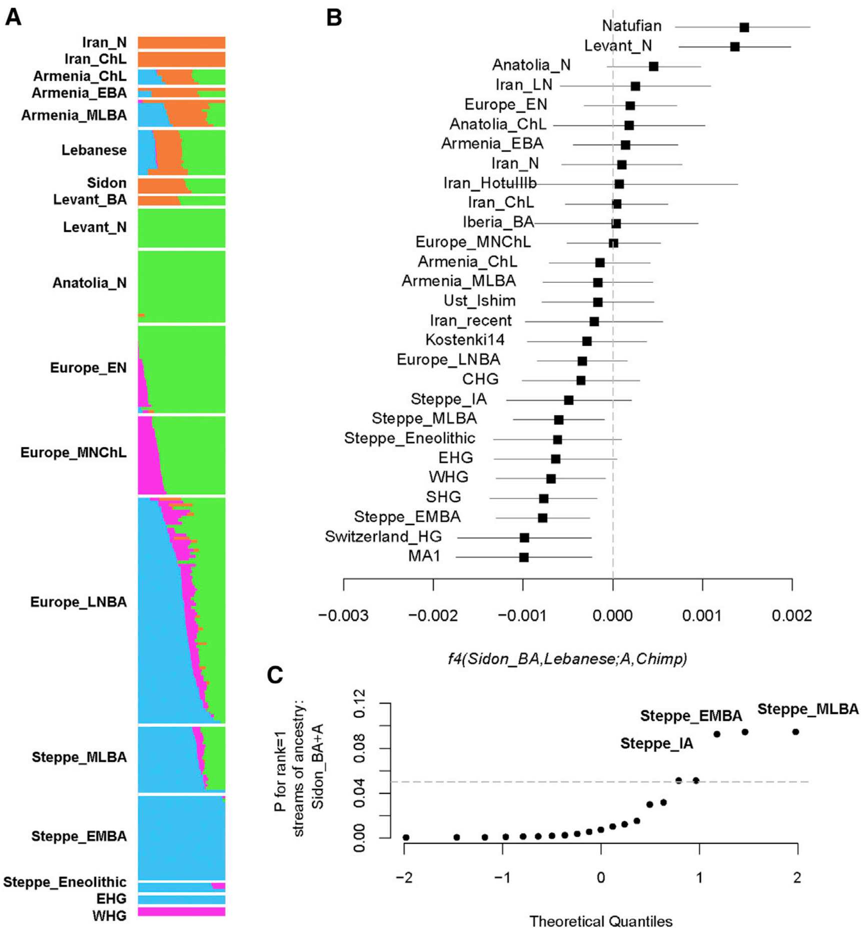

Figure 3. Admixture in Present-Day Levantine Populations

(A) Supervised ADMIXTURE using Levant_N, Iran_N, EHG, and WHG as reference populations. A Eurasian ancestry found in Eastern hunter-gatherers and the steppe Bronze Age appears in present-day Levantines after the Bronze Age.

(B) The statistic *f4*(Sidon_BA, Lebanese; Ancient Eurasian, Chimpanzee) confirms the ADMIXTURE results and is most negative for populations from the steppe and Eurasian hunter-gatherers. We show the estimated statistic value and ±3 standard errors.

(C) Present-day Lebanese can be modeled as mixture between Bronze Age Sidon and a steppe population. The model with mix proportions 0.932 ± 0.016 Sidon_BA and 0.068 ± 0.016 steppe_EMBA for Lebanese is supported with the lowest SE.

the model need not be the actual ancestral mixing populations, and the admixture could have involved a population which was itself admixed with a Steppe-like ancestry population. The time period of this mixture overlaps with the decline of the Egyptian empire and its domination over the Levant, leading some of the coastal cities to thrive, including Sidon and Tyre, which established at this time a successful maritime trade network throughout the Mediterranean. The decline in Egypt's power was also followed by a succession of conquests of the region by distant populations such as the Assyrians, Persians, and Macedonians, any or all of whom could have carried the Steppe-like ancestry observed here in the Levant after the Bronze Age.

In this report we have analyzed ancient whole-genome sequence data from a Levantine civilization and provided insights into how the Bronze Age Canaanites were related to other ancient populations and how they have contributed genetically to present-day ones (Figure 4). Many of our inferences rely on the limited number of ancient samples available, and we are only just beginning to reconstruct a genetic history of the Levant or the Near East as thoroughly as that of Europeans who, in comparison, have been extensively sampled. In the future, it will be important to examine samples from the Chalcolithic/Early Bronze Age Near East to understand the events leading to admixture between local populations and the eastern migrants. It will also be important to analyze samples from the Iron Age to trace back the Steppe-like ancestry we find today in present-day Levantines. Our current results show that such studies are feasible.

Accession Numbers

Sequencing data for 99 present-day Lebanese individuals reported in this paper are available through the European Genome-phenome Archive (EGA) under accession number EGA: EGAS00001002084. Raw sequencing reads for 5 ancient Canaanite individuals are available through the European Nucleotide Archive (ENA) under accession number ENA: PRJEB21330. Aligned sequences and genotypes can be obtained from the corresponding authors.

Supplemental Data

Supplemental Data include 14 figures and 6 tables and can be found with this article online at http://dx.doi.org/10.1016/j.ajhg.2017.06.013.

Acknowledgments

We thank the present-day donors who contributed their samples to this study and bioRxiv readers for helpful comments. M.H., Y.X., P.D., R.M., J.P.-M., M.S., and C.T.-S. were supported by The Wellcome Trust (098051).

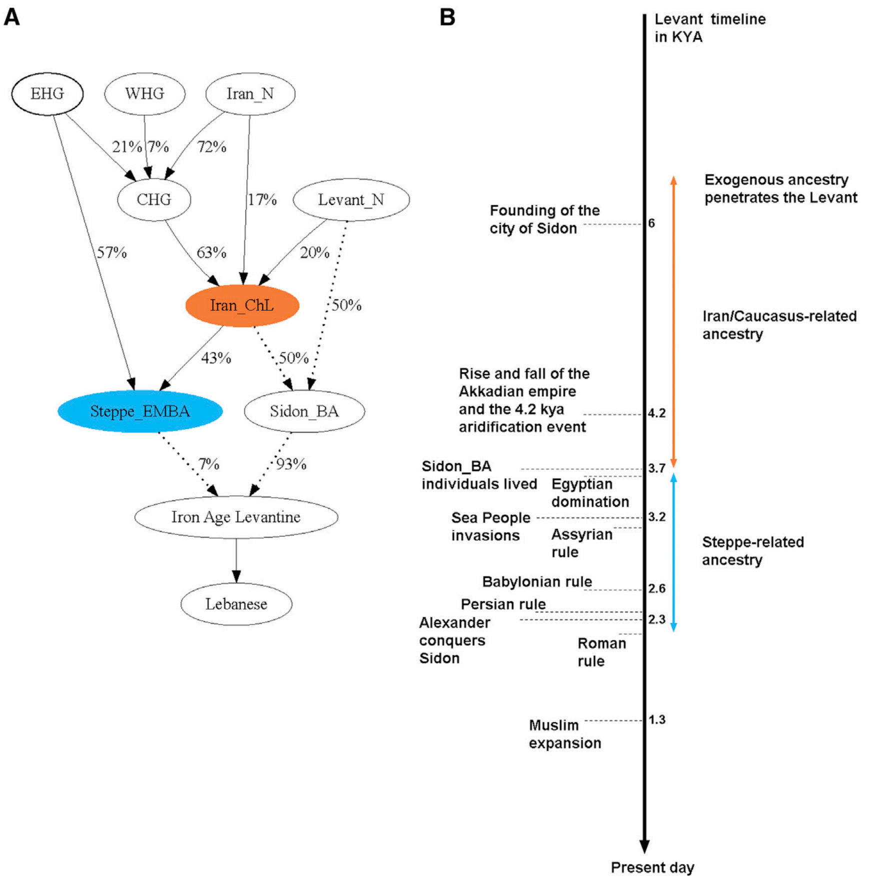

Figure 4. Genetic History of the Levant
(A) A model of population relationships which fits the *qpAdm* results from Lazaridis et al.[13] (solid arrows) and this study (dotted arrows). Percentages on arrows are the inferred admixture proportions.
(B) Levant timeline of historical events with genetically inferred admixture dates shown as colored double-ended arrows with length representing the SE.

Received: May 25, 2017
Accepted: June 27, 2017
Published: July 27, 2017

Web Resources

European Genome-phenome Archive (EGA), https://www.ebi.ac.uk/ega

European Nucleotide Archive (ENA), http://www.ebi.ac.uk/ena

References

1. Pagani, L., Schiffels, S., Gurdasani, D., Danecek, P., Scally, A., Chen, Y., Xue, Y., Haber, M., Ekong, R., Oljira, T., et al. (2015). Tracing the route of modern humans out of Africa by using 225 human genome sequences from Ethiopians and Egyptians. Am. J. Hum. Genet. *96*, 986–991.
2. Platt, D.E., Haber, M., Dagher-Kharrat, M.B., Douaihy, B., Khazen, G., Ashrafian Bonab, M., Salloum, A., Mouzaya, F., Luiselli, D., Tyler-Smith, C., et al. (2017). Mapping post-glacial expansions: the peopling of Southwest Asia. Sci. Rep. *7*, 40338.
3. Hitti, P.K. (1967). Lebanon in History: From the Earliest Times to the Present (London: Macmillan).
4. Haber, M., Gauguier, D., Youhanna, S., Patterson, N., Moorjani, P., Botigué, L.R., Platt, D.E., Matisoo-Smith, E., Soria-Hernanz, D.F., Wells, R.S., et al. (2013). Genome-wide diversity in the levant reveals recent structuring by culture. PLoS Genet. *9*, e1003316.
5. Zalloua, P.A., Platt, D.E., El Sibai, M., Khalife, J., Makhoul, N., Haber, M., Xue, Y., Izaabel, H., Bosch, E., Adams, S.M., et al.; Genographic Consortium (2008). Identifying genetic traces of historical expansions: Phoenician footprints in the Mediterranean. Am. J. Hum. Genet. *83*, 633–642.
6. Allentoft, M.E., Sikora, M., Sjögren, K.G., Rasmussen, S., Rasmussen, M., Stenderup, J., Damgaard, P.B., Schroeder, H., Ahlström, T., Vinner, L., et al. (2015). Population genomics of Bronze Age Eurasia. Nature *522*, 167–172.
7. Günther, T., Valdiosera, C., Malmström, H., Ureña, I., Rodriguez-Varela, R., Sverrisdóttir, O.O., Daskalaki, E.A., Skoglund, P., Naidoo, T., Svensson, E.M., et al. (2015). Ancient genomes link early farmers from Atapuerca in Spain to modern-day Basques. Proc. Natl. Acad. Sci. USA *112*, 11917–11922.
8. Haak, W., Lazaridis, I., Patterson, N., Rohland, N., Mallick, S., Llamas, B., Brandt, G., Nordenfelt, S., Harney, E., Stewardson, K., et al. (2015). Massive migration from the steppe was a source for Indo-European languages in Europe. Nature *522*, 207–211.
9. Lazaridis, I., Patterson, N., Mittnik, A., Renaud, G., Mallick, S., Kirsanow, K., Sudmant, P.H., Schraiber, J.G., Castellano, S., Lipson, M., et al. (2014). Ancient human genomes suggest three ancestral populations for present-day Europeans. Nature *513*, 409–413.
10. Mathieson, I., Lazaridis, I., Rohland, N., Mallick, S., Patterson, N., Roodenberg, S.A., Harney, E., Stewardson, K., Fernandes, D., Novak, M., et al. (2015). Genome-wide patterns of selection in 230 ancient Eurasians. Nature *528*, 499–503.
11. Olalde, I., Schroeder, H., Sandoval-Velasco, M., Vinner, L., Lobón, I., Ramirez, O., Civit, S., García Borja, P., Salazar-García, D.C., Talamo, S., et al. (2015). A common genetic origin

for early farmers from Mediterranean Cardial and Central European LBK cultures. Mol. Biol. Evol. *32*, 3132–3142.
12. Haber, M., Mezzavilla, M., Xue, Y., and Tyler-Smith, C. (2016). Ancient DNA and the rewriting of human history: be sparing with Occam's razor. Genome Biol. *17*, 1.
13. Lazaridis, I., Nadel, D., Rollefson, G., Merrett, D.C., Rohland, N., Mallick, S., Fernandes, D., Novak, M., Gamarra, B., Sirak, K., et al. (2016). Genomic insights into the origin of farming in the ancient Near East. Nature *536*, 419–424.
14. Gamba, C., Jones, E.R., Teasdale, M.D., McLaughlin, R.L., Gonzalez-Fortes, G., Mattiangeli, V., Domboróczki, L., Kővári, I., Pap, I., Anders, A., et al. (2014). Genome flux and stasis in a five millennium transect of European prehistory. Nat. Commun. *5*, 5257.
15. Tubb, J.N. (1998). Canaanites (London: Published for the Trustees of the British Museum by British Museum Press).
16. Markoe, G. (2000). Phoenicians (London: British Museum Press).
17. Al Khalifa, H.A.S., and Rice, M. (1986). Bahrain through the Ages: The Archaeology (London: KPI).
18. Meyer, M., and Kircher, M. (2010). Illumina sequencing library preparation for highly multiplexed target capture and sequencing. Cold Spring Harb. Protoc. *2010*, t5448.
19. Dabney, J., Knapp, M., Glocke, I., Gansauge, M.T., Weihmann, A., Nickel, B., Valdiosera, C., García, N., Pääbo, S., Arsuaga, J.L., and Meyer, M. (2013). Complete mitochondrial genome sequence of a Middle Pleistocene cave bear reconstructed from ultrashort DNA fragments. Proc. Natl. Acad. Sci. USA *110*, 15758–15763.
20. Rasmussen, M., Anzick, S.L., Waters, M.R., Skoglund, P., DeGiorgio, M., Stafford, T.W., Jr., Rasmussen, S., Moltke, I., Albrechtsen, A., Doyle, S.M., et al. (2014). The genome of a Late Pleistocene human from a Clovis burial site in western Montana. Nature *506*, 225–229.
21. Pinhasi, R., Fernandes, D., Sirak, K., Novak, M., Connell, S., Alpaslan-Roodenberg, S., Gerritsen, F., Moiseyev, V., Gromov, A., Raczky, P., et al. (2015). Optimal ancient DNA yields from the inner ear part of the human petrous bone. PLoS ONE *10*, e0129102.
22. Schubert, M., Ermini, L., Der Sarkissian, C., Jónsson, H., Ginolhac, A., Schaefer, R., Martin, M.D., Fernández, R., Kircher, M., McCue, M., et al. (2014). Characterization of ancient and modern genomes by SNP detection and phylogenomic and metagenomic analysis using PALEOMIX. Nat. Protoc. *9*, 1056–1082.
23. Garrison, E., and Marth, G. (2012). Haplotype-based variant detection from short-read sequencing. arXiv. arXiv:1207.3907 [q-bio.GN]. https://arxiv.org/abs/1207.3907.
24. Stamatakis, A. (2014). RAxML version 8: a tool for phylogenetic analysis and post-analysis of large phylogenies. Bioinformatics *30*, 1312–1313.
25. Letunic, I., and Bork, P. (2016). Interactive tree of life (iTOL) v3: an online tool for the display and annotation of phylogenetic and other trees. Nucleic Acids Res. *44* (W1), W242–W245.
26. Korneliussen, T.S., Albrechtsen, A., and Nielsen, R. (2014). ANGSD: Analysis of Next Generation Sequencing Data. BMC Bioinformatics *15*, 356.
27. Rasmussen, M., Guo, X., Wang, Y., Lohmueller, K.E., Rasmussen, S., Albrechtsen, A., Skotte, L., Lindgreen, S., Metspalu, M., Jombart, T., et al. (2011). An Aboriginal Australian genome reveals separate human dispersals into Asia. Science *334*, 94–98.
28. Renaud, G., Slon, V., Duggan, A.T., and Kelso, J. (2015). Schmutzi: estimation of contamination and endogenous mitochondrial consensus calling for ancient DNA. Genome Biol. *16*, 224.
29. Skoglund, P., Northoff, B.H., Shunkov, M.V., Derevianko, A.P., Pääbo, S., Krause, J., and Jakobsson, M. (2014). Separating endogenous ancient DNA from modern day contamination in a Siberian Neandertal. Proc. Natl. Acad. Sci. USA *111*, 2229–2234.
30. Skoglund, P., Posth, C., Sirak, K., Spriggs, M., Valentin, F., Bedford, S., Clark, G.R., Reepmeyer, C., Petchey, F., Fernandes, D., et al. (2016). Genomic insights into the peopling of the Southwest Pacific. Nature *538*, 510–513.
31. Haber, M., Mezzavilla, M., Bergström, A., Prado-Martinez, J., Hallast, P., Saif-Ali, R., Al-Habori, M., Dedoussis, G., Zeggini, E., Blue-Smith, J., et al. (2016). Chad genetic diversity reveals an African history marked by multiple Holocene eurasian migrations. Am. J. Hum. Genet. *99*, 1316–1324.
32. The 1000 Genomes Project Consortium (2015). A global reference for human genetic variation. Nature *526*, 68–74.
33. Wigginton, J.E., Cutler, D.J., and Abecasis, G.R. (2005). A note on exact tests of Hardy-Weinberg equilibrium. Am. J. Hum. Genet. *76*, 887–893.
34. Delaneau, O., Marchini, J., and Zagury, J.F. (2011). A linear complexity phasing method for thousands of genomes. Nat. Methods *9*, 179–181.
35. Browning, B.L., and Browning, S.R. (2016). Genotype imputation with millions of reference samples. Am. J. Hum. Genet. *98*, 116–126.
36. Jones, E.R., Gonzalez-Fortes, G., Connell, S., Siska, V., Eriksson, A., Martiniano, R., McLaughlin, R.L., Gallego Llorente, M., Cassidy, L.M., Gamba, C., et al. (2015). Upper Palaeolithic genomes reveal deep roots of modern Eurasians. Nat. Commun. *6*, 8912.
37. Fu, Q., Li, H., Moorjani, P., Jay, F., Slepchenko, S.M., Bondarev, A.A., Johnson, P.L., Aximu-Petri, A., Prüfer, K., de Filippo, C., et al. (2014). Genome sequence of a 45,000-year-old modern human from western Siberia. Nature *514*, 445–449.
38. Raghavan, M., Skoglund, P., Graf, K.E., Metspalu, M., Albrechtsen, A., Moltke, I., Rasmussen, S., Stafford, T.W., Jr., Orlando, L., Metspalu, E., et al. (2014). Upper Palaeolithic Siberian genome reveals dual ancestry of Native Americans. Nature *505*, 87–91.
39. Patterson, N., Moorjani, P., Luo, Y., Mallick, S., Rohland, N., Zhan, Y., Genschoreck, T., Webster, T., and Reich, D. (2012). Ancient admixture in human history. Genetics *192*, 1065–1093.
40. Patterson, N., Price, A.L., and Reich, D. (2006). Population structure and eigenanalysis. PLoS Genet. *2*, e190.
41. Nakouzi, G., Kreidieh, K., and Yazbek, S. (2015). A review of the diverse genetic disorders in the Lebanese population: highlighting the urgency for community genetic services. J. Community Genet. *6*, 83–105.
42. Hager, J., Kamatani, Y., Cazier, J.B., Youhanna, S., Ghassibe-Sabbagh, M., Platt, D.E., Abchee, A.B., Romanos, J., Khazen, G., Othman, R., et al.; FGENTCARD Consortium (2012). Genome-wide association study in a Lebanese cohort confirms PHACTR1 as a major determinant of coronary artery stenosis. PLoS ONE *7*, e38663.
43. Ghassibe-Sabbagh, M., Haber, M., Salloum, A.K., Al-Sarraj, Y., Akle, Y., Hirbli, K., Romanos, J., Mouzaya, F., Gauguier, D., Platt, D.E., et al. (2014). T2DM GWAS in the Lebanese

population confirms the role of TCF7L2 and CDKAL1 in disease susceptibility. Sci. Rep. *4*, 7351.

44. Li, Y., Vinckenbosch, N., Tian, G., Huerta-Sanchez, E., Jiang, T., Jiang, H., Albrechtsen, A., Andersen, G., Cao, H., Korneliussen, T., et al. (2010). Resequencing of 200 human exomes identifies an excess of low-frequency non-synonymous coding variants. Nat. Genet. *42*, 969–972.
45. Poznik, G.D. (2016). Identifying Y-chromosome haplogroups in arbitrarily large samples of sequenced or genotyped men. bioRxiv. http://dx.doi.org/10.1101/088716.
46. Chiaroni, J., King, R.J., Myres, N.M., Henn, B.M., Ducourneau, A., Mitchell, M.J., Boetsch, G., Sheikha, I., Lin, A.A., Nik-Ahd, M., et al. (2010). The emergence of Y-chromosome haplogroup J1e among Arabic-speaking populations. Eur. J. Hum. Genet. *18*, 348–353.
47. Al-Zahery, N., Pala, M., Battaglia, V., Grugni, V., Hamod, M.A., Hooshiar Kashani, B., Olivieri, A., Torroni, A., Santachiara-Benerecetti, A.S., and Semino, O. (2011). In search of the genetic footprints of Sumerians: a survey of Y-chromosome and mtDNA variation in the Marsh Arabs of Iraq. BMC Evol. Biol. *11*, 288.
48. Semino, O., Magri, C., Benuzzi, G., Lin, A.A., Al-Zahery, N., Battaglia, V., Maccioni, L., Triantaphyllidis, C., Shen, P., Oefner, P.J., et al. (2004). Origin, diffusion, and differentiation of Y-chromosome haplogroups E and J: inferences on the neolithization of Europe and later migratory events in the Mediterranean area. Am. J. Hum. Genet. *74*, 1023–1034.
49. Weissensteiner, H., Forer, L., Fuchsberger, C., Schöpf, B., Kloss-Brandstätter, A., Specht, G., Kronenberg, F., and Schönherr, S. (2016). mtDNA-Server: next-generation sequencing data analysis of human mitochondrial DNA in the cloud. Nucleic Acids Res. *44* (W1), W64–W69.
50. Loh, P.R., Lipson, M., Patterson, N., Moorjani, P., Pickrell, J.K., Reich, D., and Berger, B. (2013). Inferring admixture histories of human populations using linkage disequilibrium. Genetics *193*, 1233–1254.
51. Cullen, H.M., deMenoca, P.B., Hemming, S., Hemming, G., Brown, F.H., Guilderson, T., and Sirocko, F. (2000). Climate change and the collapse of the Akkadian empire: Evidence from the deep sea. Geology *28*, 4.
52. deMenocal, P.B. (2001). Cultural responses to climate change during the late Holocene. Science *292*, 667–673.
53. Weiss, H., Courty, M.A., Wetterstrom, W., Guichard, F., Senior, L., Meadow, R., and Curnow, A. (1993). The genesis and collapse of third millennium north mesopotamian civilization. Science *261*, 995–1004.
54. Alexander, D.H., Novembre, J., and Lange, K. (2009). Fast model-based estimation of ancestry in unrelated individuals. Genome Res. *19*, 1655–1664.

The American Journal of Human Genetics, Volume *101*

Supplemental Data

Continuity and Admixture in the Last Five Millennia of Levantine History from Ancient Canaanite and Present-Day Lebanese Genome Sequences

Marc Haber, Claude Doumet-Serhal, Christiana Scheib, Yali Xue, Petr Danecek, Massimo Mezzavilla, Sonia Youhanna, Rui Martiniano, Javier Prado-Martinez, Michał Szpak, Elizabeth Matisoo-Smith, Holger Schutkowski, Richard Mikulski, Pierre Zalloua, Toomas Kivisild, and Chris Tyler-Smith

Supplementary materials

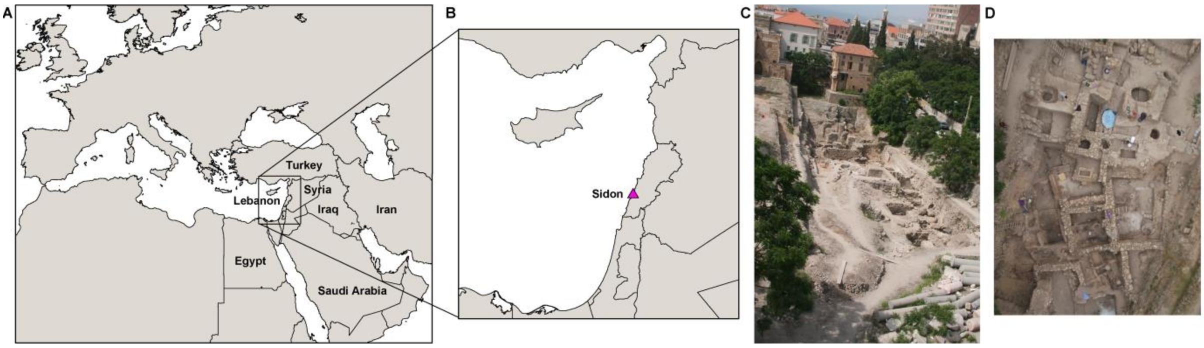

Figure S1. The Sidon excavation site. (A) Map shows the location of Lebanon with present-day political borders in the Near East. (B) A magnification showing the Levant region and the location of the city of Sidon. (C) Photo shows the Sidon excavation site which included the burials of individuals studied here. (D) Aerial view of excavation details from Season 12 (Bronze Age structures).

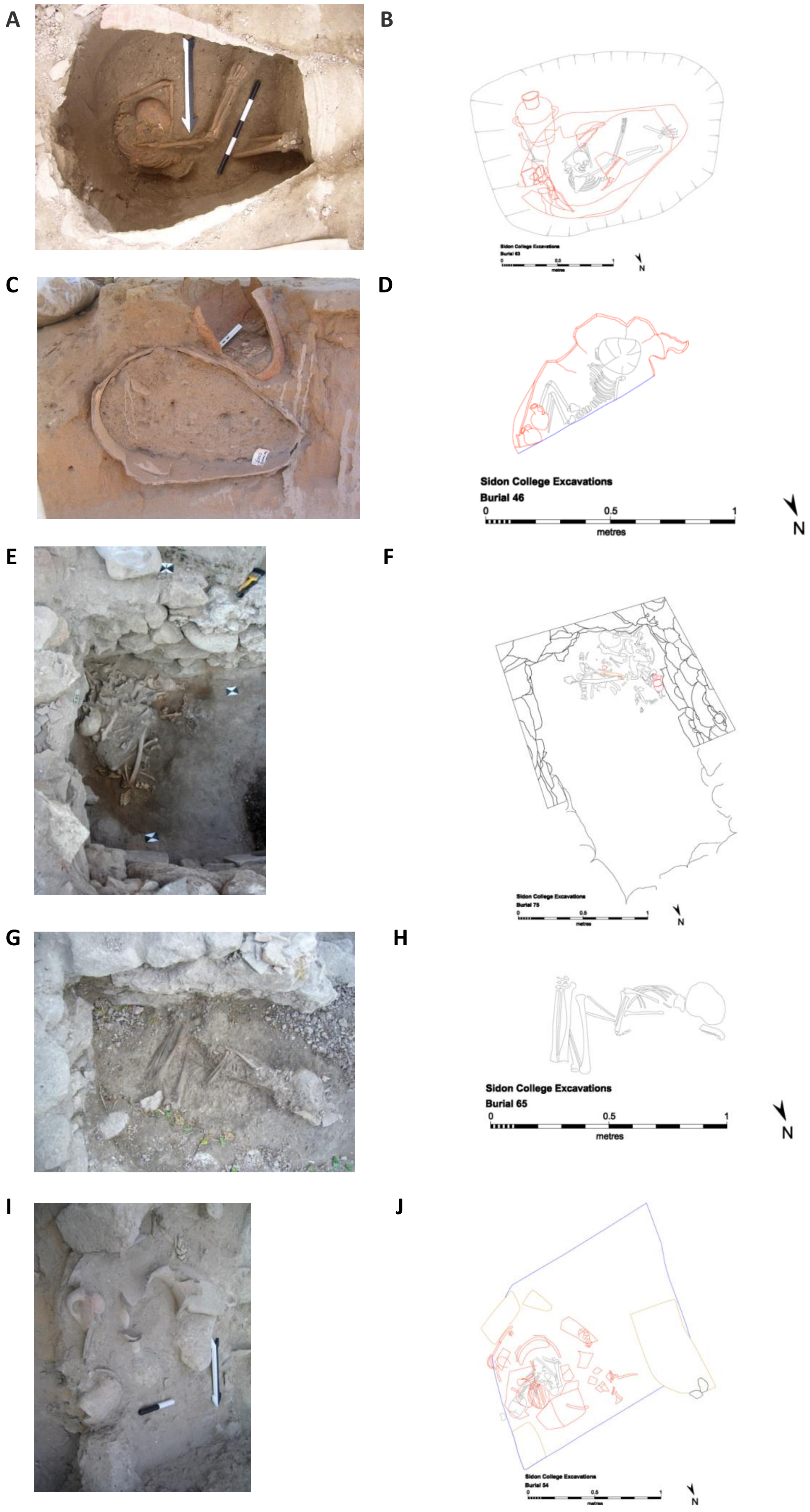

A
B
Sidon College Excavations
Burial 63
0
0.5
1
metres
N
C
D
Sidon College Excavations
Burial 46
0
0.5
1
metres
N
E
F
Sidon College Excavations
Burial 75
0
0.5
1
metres
N
G
H
Sidon College Excavations
Burial 65
0
0.5
1
metres
N
I
J
Sidon College Excavations
Burial 54
0
0.5
1
metres
N

Figure S2. Burials of individuals analysed in this study. (A) and (B) burial 63: Stratum 6 Middle Bronze Age II B about 1600 BC. Consisted of an unusually large burial containing the remains of at least two individuals. (C) and (D) burial 46: Intermediate Middle Bronze Age II A-B about 1750 BC. This burial consisted of a jar burial containing an infant. (E) and (F) burial 75: Intermediate Middle Bronze Age II A-B about 1750 BC. It contained the commingled remains of 3 individuals. (G) and (H) burial 65: Stratum 6 Middle Bronze Age II B about 1600 BC. This burial consisted of a simple unlined burial of a single sub adult individual. (I) and (J) burial 54: Middle Bronze Age II B around about 1650 BC. This jar consisted of the burial of a sub adult, probably 8-12 years old.

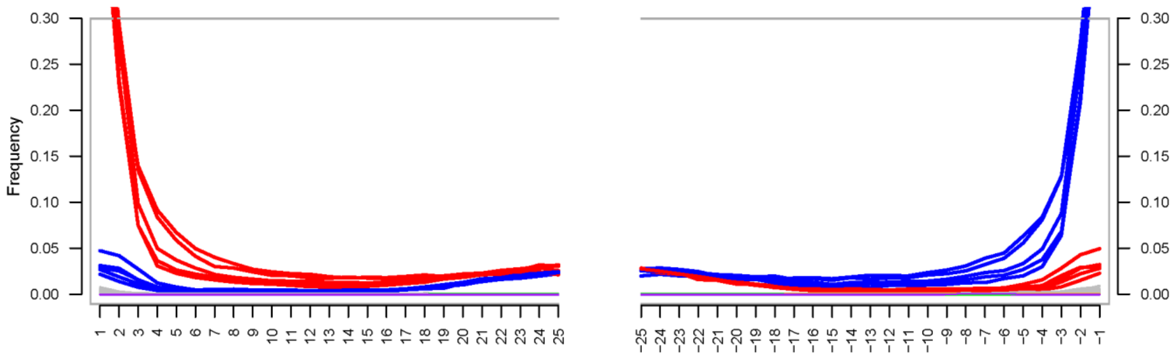

Figure S3. Post-mortem damage patterns. Base substitutions C>T from the 5" (left) and G>A from the 3" end (right) for all Sidon samples show patterns typical of ancient DNA damage.

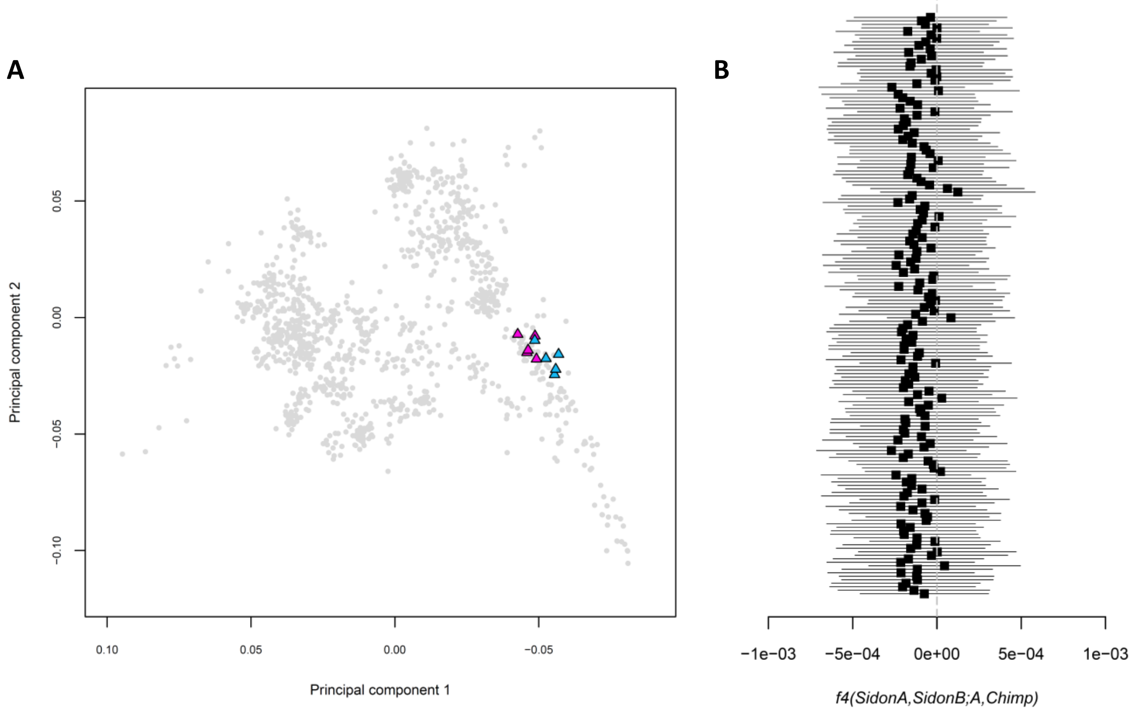

Figure S4. Testing for contamination using sequences with evidence of post-mortem damage (PMD). (A) PCA is similar to Figure 1 but additionally includes the ancient individuals represented only by sequences with a PMD score of at least 3 (blue triangles) to remove potentially contaminating sequences from present-day individuals. The samples overlap samples with no damage-restriction (pink triangles). (B) *f4* statistics testing if a modern population *A* in the dataset has excess affinity to samples with no damage-restriction (*SidonB*) compared with *SidonA* represented only by sequences with a PMD score >3. Tests for all modern populations do not significantly deviate from zero. The PCA and *f4* statistics suggest the Sidon DNA samples are authentic and minimally contaminated.

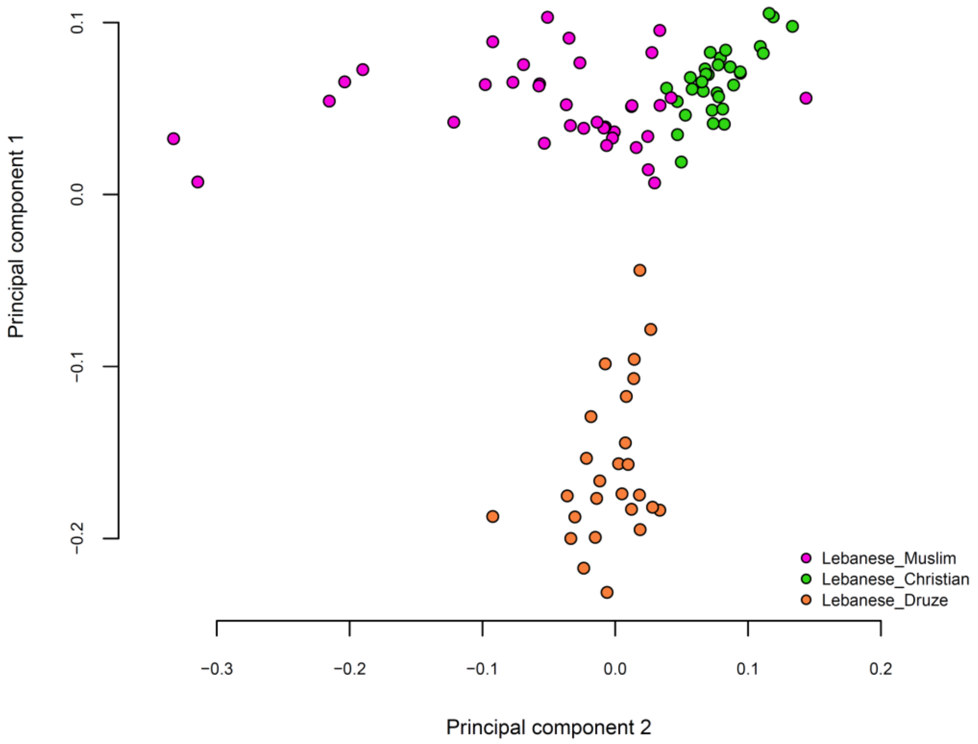

Figure S5. Genetic structure in present-day Lebanese. Principal components analysis of 99 present-day Lebanese sequenced in this study. We used the *smartpca* program of EIGENSOFT on SNPs overlapping with the 1.2M SNPs from Lazaridis et al. 2016[1] and set killr2: YES, r2thresh: 0.7, outliersigmathresh: 5. The genetic diversity of the sequenced Lebanese captures the previously-described genetic diversity in Lebanon[2] using a set with a larger sample size (1341) and array data.

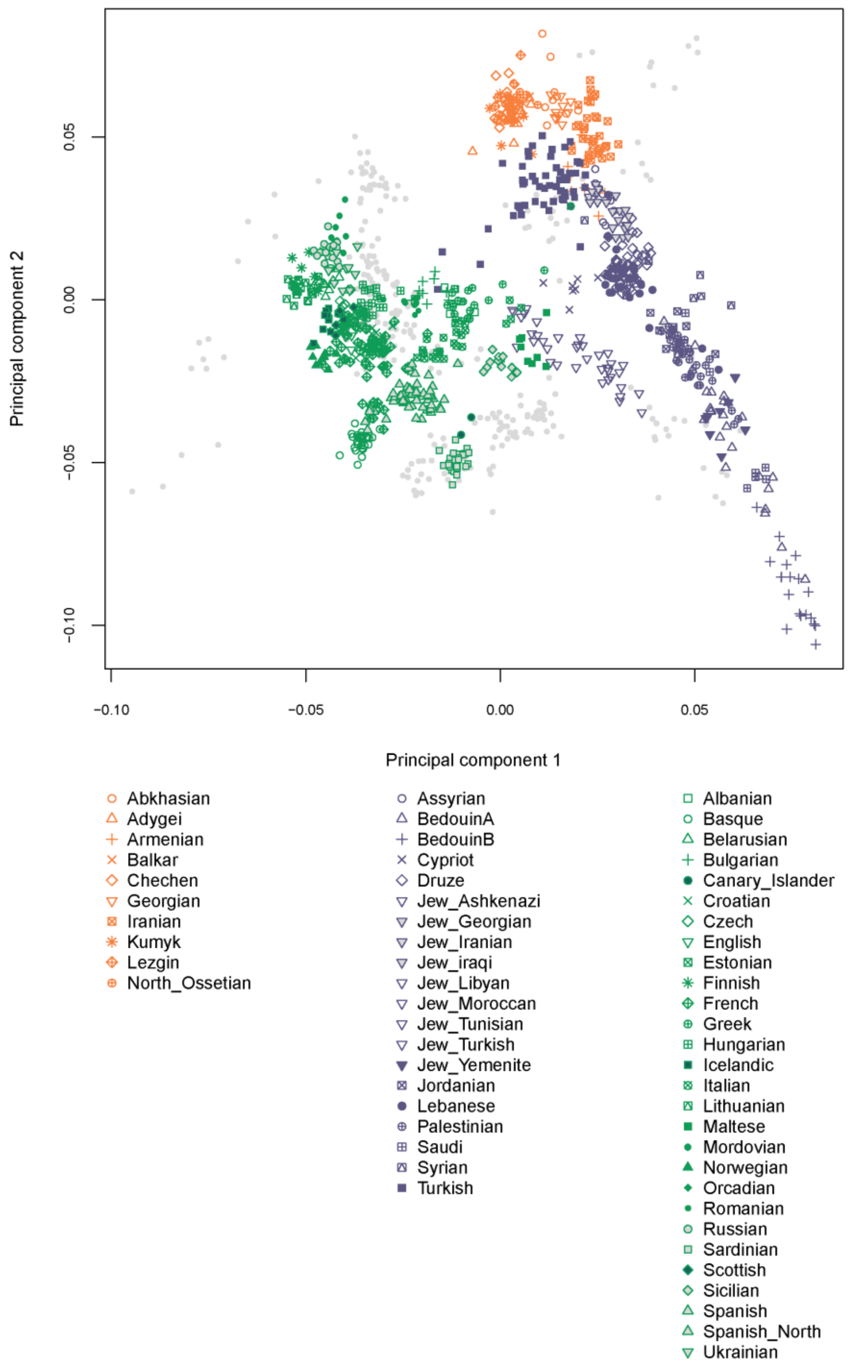

Figure S6. PCA showing the position of present-day populations. The gray points represent the projected ancient samples shown in Figure1.

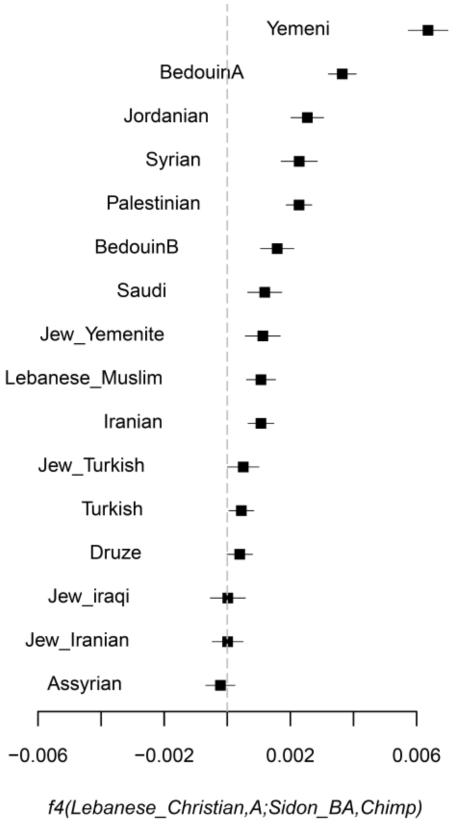

Figure S7. Testing Sidon_BA affinity to present-day Near Easterners. The statistic *f4*(Lebanese_Christian, *Test*; Sidon_BA, Chimpanzee) is significantly positive for most populations when *Test* is a present-day Near Easterner, indicating Sidon_BA shares more alleles with the Lebanese than with other Near Easterners. We chose Lebanese_Christian to represent present-day Lebanese in this test as this population has been shown to be relatively isolated and had no significant admixture in recent times with neighbouring populations. In this and following figures we plot the *f4* statistic value and ±3 standard errors.

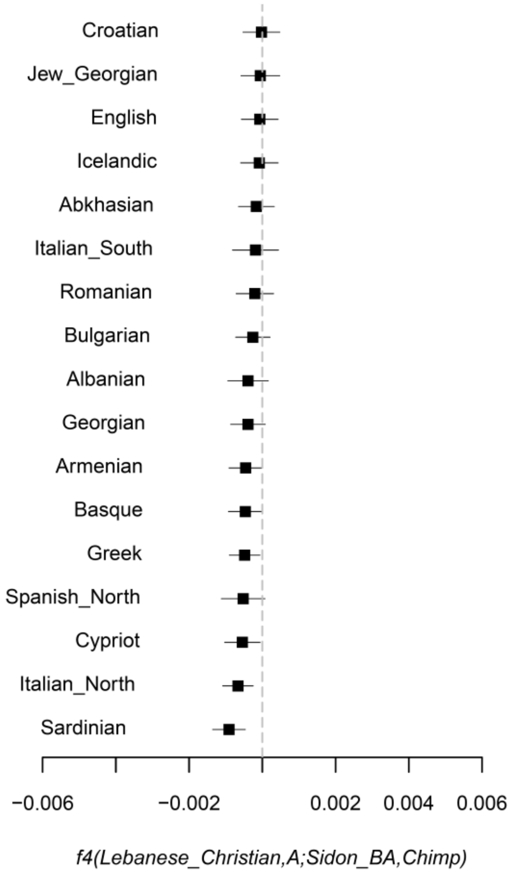

Figure S8. Testing Sidon_BA affinity to worldwide populations. The plot shows all the negative statistics from *f4*(Lebanese_Christian, *Test*; Sidon_BA, Chimpanzee) when *Test* is one of the 150 present-day populations available in the Human Origins dataset.

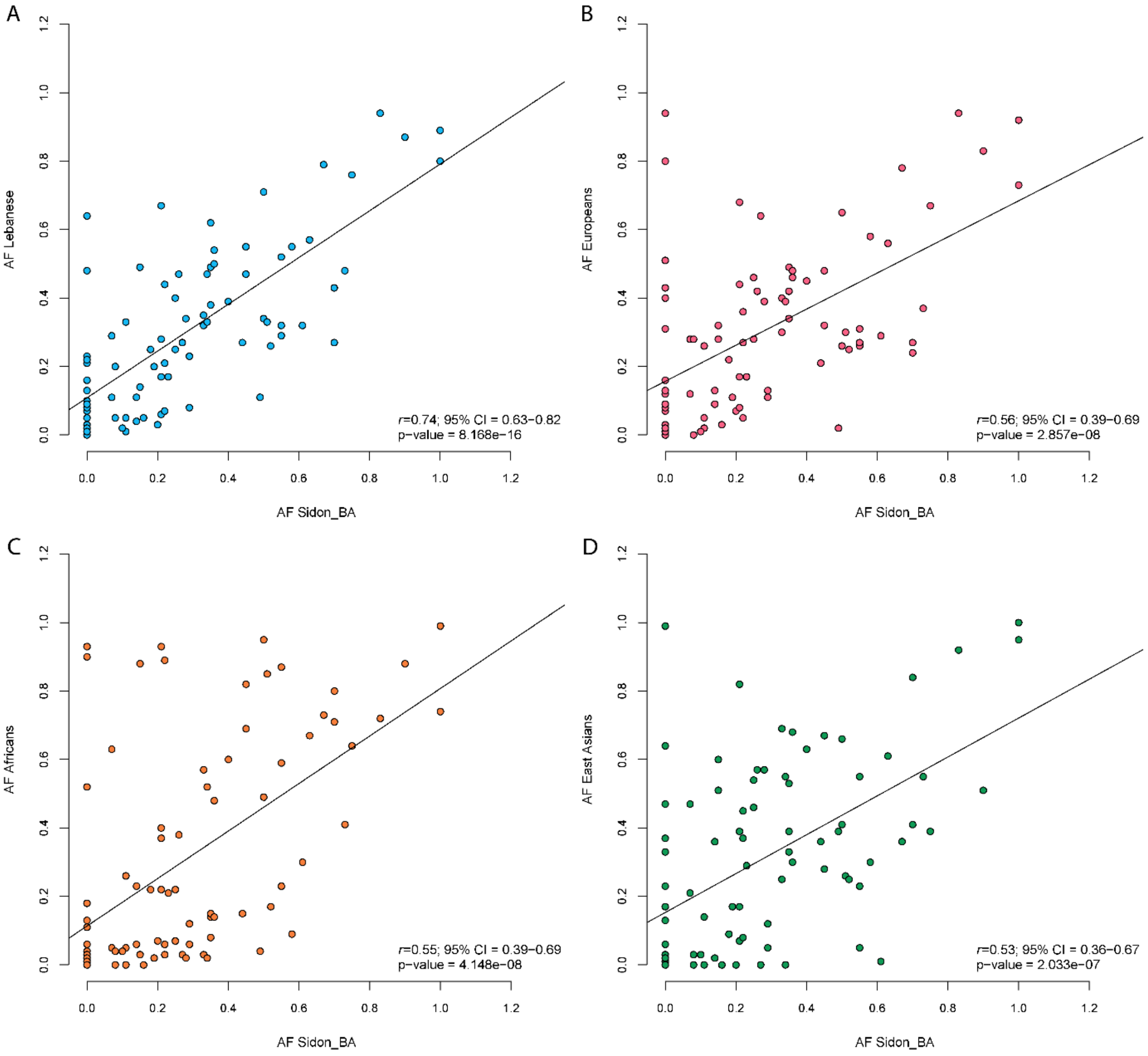

Figure S9. Correlation between the frequencies of the putative functional variants in Sidon_BA and present-day populations: (A) Lebanese (B) Europeans (C) Africans and (D) East Asians.

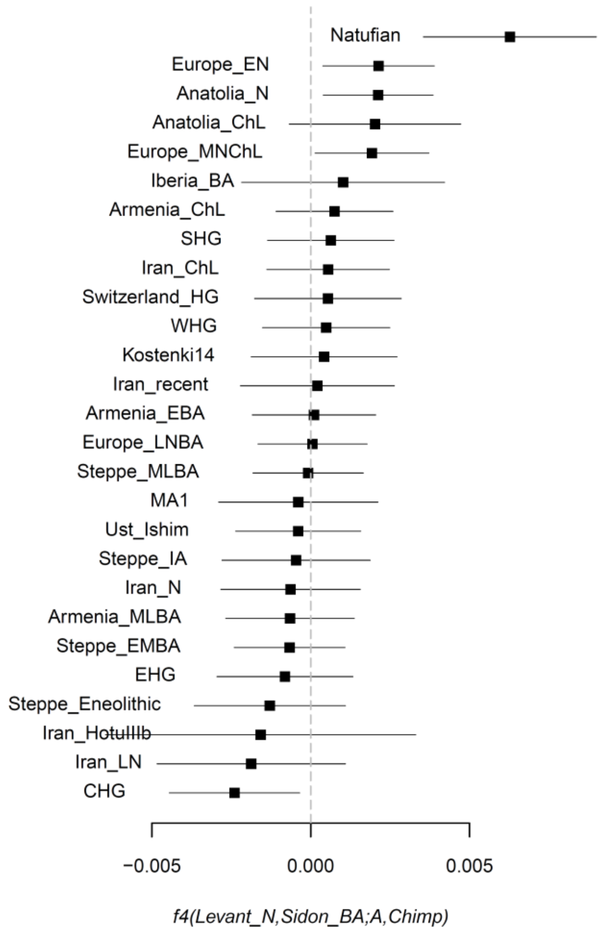

Figure S10. Admixture in Bronze Age Levantine populations (transversions only). We replicate the statistic *f4*(Levant_N, Sidon_BA; Ancient Eurasian, Chimpanzee) using ~120,000 transversions and get results qualitatively similar to the results from using all variants.

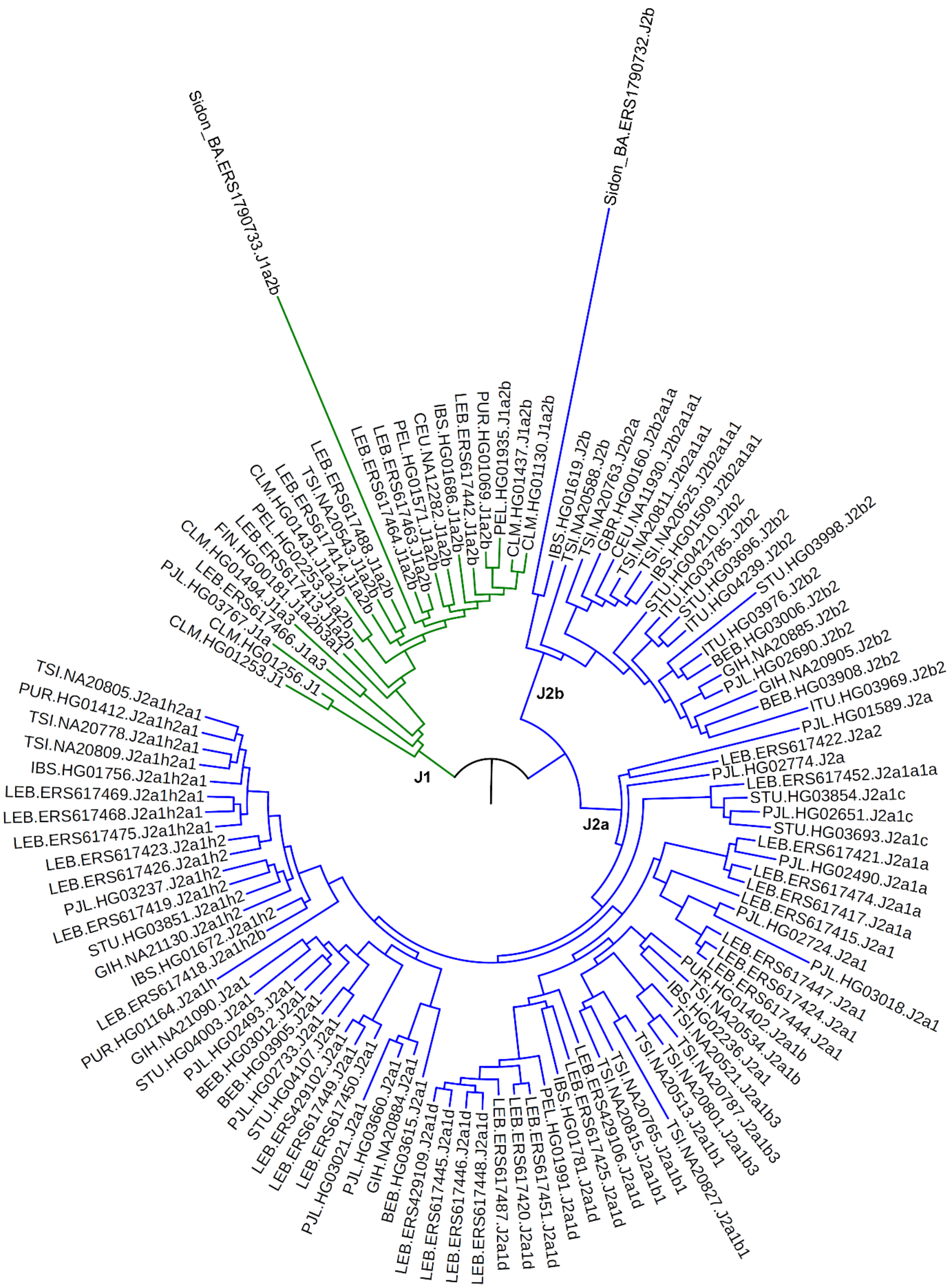

Figure S11. Y-chromosome J lineage relationship tree. A maximum likelihood phylogeny of Y chromosomes belonging to haplogroup J inferred using RAxML on males from the 1000 Genomes Project (76), present-day Lebanese (35), and Sidon_BA (2). Tip labels show population and sample name as well as haplogroup identified by yHaplo using ISOGG 2016.01.04 annotations.

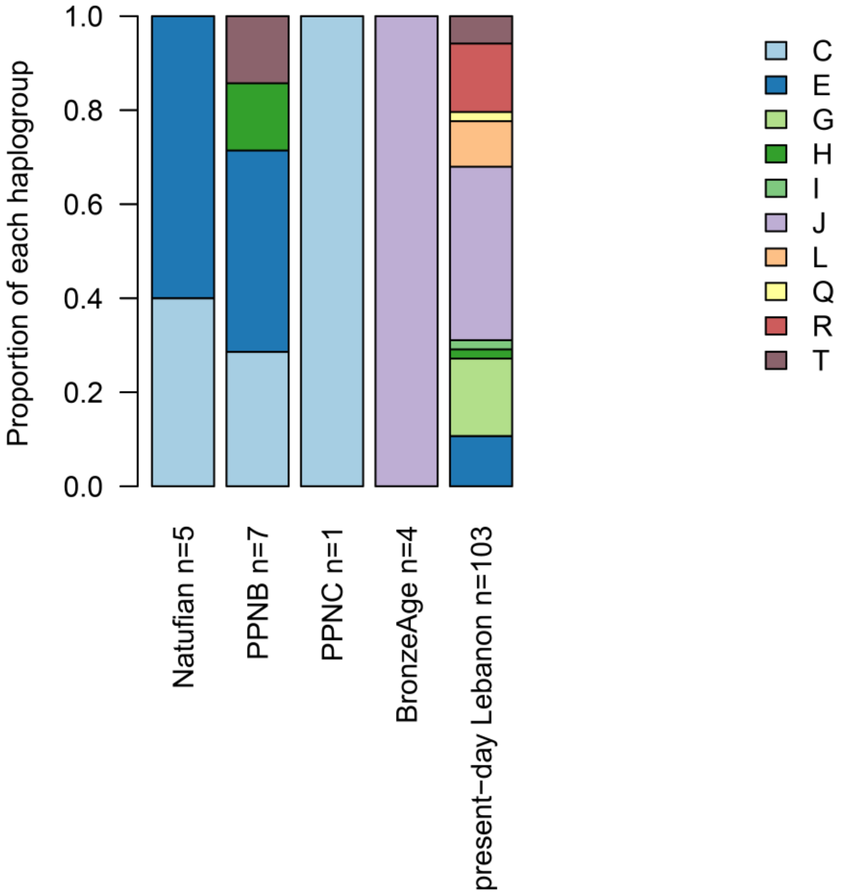

Figure S12. Y-chromosome lineages frequencies in the Levant from Natufians to present-day populations.

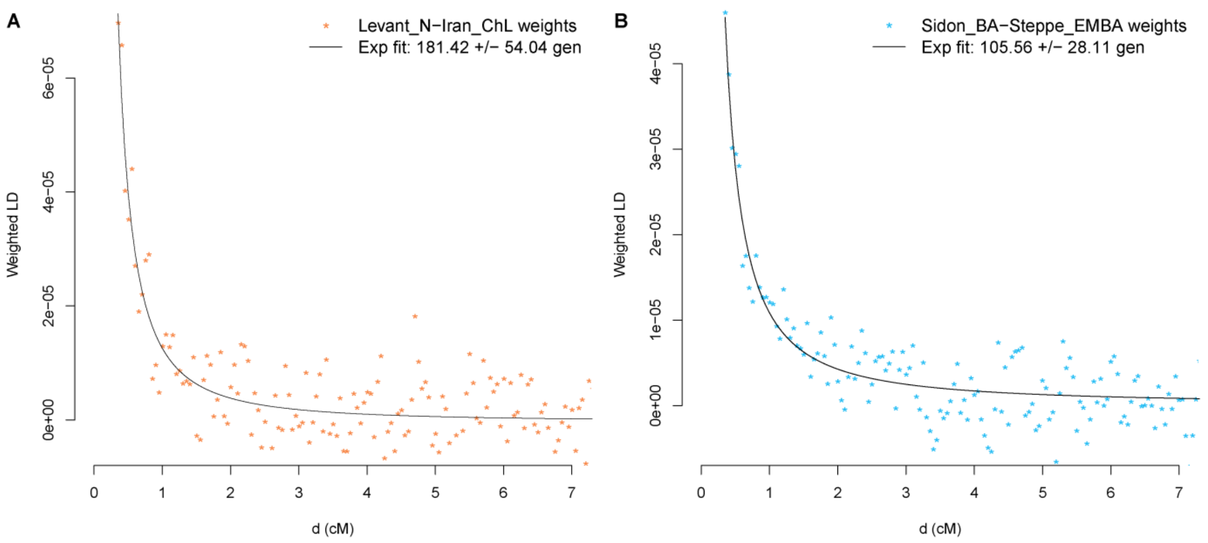

Figure S13. Time of admixture in the Levant. (A) and (B) Weighted LD decay curves for present-day Lebanese using ancient populations as references. (A) Using a generation time of 28 years; a mixture between populations related to Neolithic Levant and Chalcolithic Iran appears to have occurred 6,600-3,550 years ago and (B) mixture between populations related to Bronze Age Sidon and Bronze Age steppe occurred 3,750-2,170 years ago.

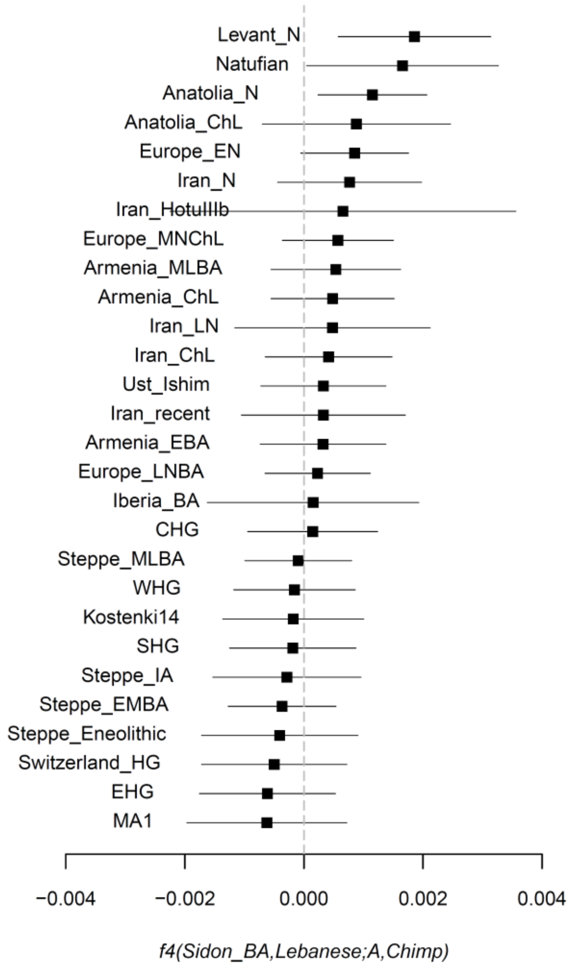

Figure S14. Admixture in present-day Levantine populations (transversions only). We replicate the statistic *f4*(Sidon_BA, Lebanese; Ancient Eurasian, Chimpanzee) using only transversions and get results qualitatively similar to the results from using all variants.

Table S1. Contamination estimates.

Sample	From mtDNA analysis (CI)[a]	From male X chromosome analysis (SE)[b]
**ERS1790732**	0.01 (0-0.02)	
Method1[†]		0.012 (0.002)
Method2		0.014 (0.003)
**ERS1790733**	0.01 (0-0.02)	
Method1		0.017 (0.005)
Method2		0.019 (0.005)
**ERS1790729**	0.01 (0-0.02)	
**ERS1790730**	0.01 (0-0.02)	
**ERS1790731**	0.01 (0-0.02)	

[a]Using schmutzi[3]
[b]Two tests using ANGSD based on methods described in Rasmussen et al. 2011 [4]

Table S2. Phenotypic and disease traits in Sidon_BA. List of putatively functional variants previously identified in the Levant region and their allele frequencies in present-day populations as well as Bronze Age Sidon. We include the read counts as guidance to allele authenticity: for example little supported C>T and G>A variations should be taken with caution.

							AF in present-day populations				AF in Bronze Age Sidon	Read counts in ancient samples				Sidon samples with coverage
Chr	Position	rs ID	Ref	Alt	Gene	Phenotype	AF_Lebanese	AF_AFR	AF_ASN	AF_EUR	AF_Sidon	total A	total C	total G	total T	n
2	136608646	rs4988235	G	A	MCM6	Lactose tolerance	0.02	0.03	0	0.51	0.00	0	0	6	0	3
5	33951693	rs16891982	C	G	SLC45A2	Pigmentation	0.64	0.04	0.01	0.94	0.00	0	9	0	0	4
5	33963870	rs26722	C	T	SLC45A2	Pigmentation	0.11	0.04	0.39	0.02	0.49	0	7	0	5	4
15	28365618	rs12913832	A	G	HERC2	Pigmentation	0.27	0.03	0	0.64	0.27	14	0	3	0	4
15	48426484	rs1426654	A	G	SLC24A5	Pigmentation	0.10	0.93	0.99	0	0.00	16	0	0	0	4
4	72618334	rs7041	A	C	GC	Vitamin D levels	0.55	0.09	0.3	0.58	0.58	3	7	0	0	5
11	71152258	rs736894	C	T	DHCR7	Vitamin D levels	0.34	0.49	0.41	0.26	0.50	0	6	0	4	4
7	141672604	rs10246939	T	C	TAS2R38	Bitter taste perception	0.54	0.48	0.68	0.46	0.36	0	5	0	10	5
9	94118258	rs10991898	C	T	AUH	3-Methylglutaconic aciduria type I	0.20	0.02	0.17	0.11	0.19	0	5	0	2	4
4	100504566	rs61733139	G	C	MTTP	Abetalipoproteinemia	0.01	0.05	0	0.05	0.11	1	0	8	0	5
4	100504664	rs3816873	T	C	MTTP	Abetalipoproteinemia	0.33	0.26	0.14	0.26	0.11	0	1	0	10	4
4	100516022	rs2306985	C	G	MTTP	Abetalipoproteinemia	0.47	0.82	0.67	0.32	0.45	0	3	0	1	3
4	1803704	rs2234909	T	C	FGFR3	Achondroplasia	0.17	0.37	0.07	0.17	0.21	0	1	0	5	4
11	5247141	rs1609812	G	A	HBB	Beta thalassemia major	0.87	0.88	0.51	0.83	0.90	10	0	2	0	5
11	5248050	rs35004220	C	T	HBB	Beta thalassemia major	0.05	0	0	0	0.08	0	10	0	1	4
15	91326099	rs2227935	C	T	BLM	Bloom syndrome	0.03	0.07	0	0.07	0.20	0	8	0	2	5
17	41244936	rs799917	G	A	BRCA1	Breast-ovarian cancer	0.44	0.89	0.37	0.36	0.22	4	0	11	0	5
6	12903957	rs9349379	A	G	PHACTR1	CAD	0.35	0.03	0.69	0.4	0.33	3	0	2	0	4
9	22098574	rs4977574	A	G	CDKN2B-AS1	CAD	0.62	0.14	0.53	0.49	0.35	5	0	3	0	4
9	107620867	rs2230806	C	T	ABCA1	CAD in familial hypercholesterolemia, protection against	0.43	0.71	0.41	0.24	0.70	0	5	0	11	5
1	53712727	rs5174	C	T	LRP8	Myocardial infarction	0.21	0.02	0.03	0.4	0.00	0	6	0	0	3
6	31540313	rs909253	A	G	LTA	Myocardial infarction	0.23	0.52	0.47	0.31	0.00	11	0	0	0	4
12	10313448	rs11053646	C	G	OLR1	Myocardial infarction	0.06	0.22	0.17	0.08	0.21	0	9	2	1	5
14	35761675	rs1048990	C	G	PSMA6	Myocardial infarction	0.22	0.03	0.37	0.16	0.00	0	6	0	0	3
17	45368337	rs15908	A	C	ITGB3	Myocardial infarction	0.48	0.41	0.55	0.37	0.73	3	8	0	0	4
13	113772707	rs6041	G	A	F7	Myocardial infarction, decreasedsusceptibility to	0.23	0.12	0.05	0.11	0.29	2	0	4	0	4
6	6174866	rs5982	G	A	F13A1	Myocardial infarction, protection against	0.27	0.15	0.36	0.21	0.44	3	0	4	0	3
12	121416650	rs1169288	A	C	HNF1A	Diabetes mellitus, noninsulin-dependent, 2	0.49	0.08	0.39	0.34	0.35	3	2	0	0	3
12	121416864	rs1800574	C	T	HNF1A	Diabetes mellitus, noninsulin-dependent, 2	0.05	0	0	0.03	0.16	0	8	0	1	5
4	6302519	rs1801212	G	A	WFS1	Diabetes mellitus, noninsulin-dependent, association with	0.80	0.99	1	0.73	1.00	14	0	0	0	4
4	6302889	rs1801208	G	A	WFS1	Diabetes mellitus, noninsulin-dependent, association with	0.07	0.03	0.08	0.05	0.22	1	0	5	0	4
7	44189393	rs144723656	G	A	GCK	Diabetes mellitus, noninsulin-dependent, late onset	0.00	0	0	0.01	0.00	0	0	12	0	4
8	118184783	rs13266634	C	T	SLC30A8	Diabetes mellitus, noninsulin-dependent, susceptibility to	0.25	0.07	0.46	0.28	0.25	0	13	0	3	4
2	182542998	rs8192556	G	T	NEUROD1	T2D	0.05	0	0	0.02	0.11	1	0	17	0	4
2	227660544	rs1801278	C	T	IRS1	T2D	0.04	0.06	0.02	0.09	0.14	0	4	0	1	3
3	185511687	rs4402960	G	T	IGF2BP2	T2D	0.32	0.57	0.25	0.3	0.33	1	0	3	3	3
3	185529080	rs1470579	A	C	IGF2BP2	T2D	0.33	0.85	0.26	0.3	0.51	6	6	0	0	5
6	20679709	rs7756992	A	G	CDKAL1	T2D	0.29	0.63	0.47	0.28	0.07	13	0	1	0	4
7	127251188	rs712701	T	G	PAX4	T2D	0.79	0.73	0.36	0.78	0.67	1	0	5	2	4
10	114808902	rs12255372	G	T	TCF7L2	T2D	0.32	0.3	0.01	0.29	0.61	0	0	6	9	4
11	2857194	rs2237895	A	C	KCNQ1	T2D	0.38	0.15	0.33	0.42	0.35	3	2	0	0	2
11	17408630	rs5215	C	T	KCNJ11	T2D	0.71	0.95	0.66	0.65	0.50	0	3	0	2	4
11	17409069	rs5218	G	A	KCNJ11	T2D	0.14	0.03	0.51	0.32	0.15	2	0	9	0	4
11	44280090	rs729287	C	T	ALX4	T2D	0.21	0.06	0.45	0.27	0.22	0	10	0	4	4
17	36047417	rs3110641	G	A	HNF1B	T2D	0.32	0.59	0.23	0.26	0.55	3	0	5	0	4
17	36098040	rs4430796	A	G	HNF1B	T2D	0.55	0.69	0.28	0.48	0.45	9	0	7	0	4
20	43058267	rs147638455	A	G	HNF4A	T2D	0.01	0	0	0	0.00	7	0	0	0	4
3	10328453	rs4684677	T	A	GHRL	Obesity	0.03	0.01	0.01	0.07	0.00	0	0	0	5	3
5	148206440	rs1042713	G	A	ADRB2	Obesity	0.47	0.52	0.55	0.39	0.34	2	0	5	0	4
5	148206885	rs1800888	C	T	ADRB2	Obesity	0.02	0	0	0.02	0.00	0	7	0	0	3
5	149212243	rs7732671	G	C	PPARGC1B	Obesity	0.13	0.18	0.06	0.08	0.00	0	0	5	0	3
6	132212694	rs7754561	A	G	ENPP1	Obesity	0.52	0.87	0.55	0.27	0.55	2	0	4	0	3
8	37823798	rs4994	A	G	ADRB3	Obesity	0.07	0.9	0.13	0.8	0.00	12	0	0	0	4
16	53800954	rs1421085	T	C	FTO	Obesity	0.48	0.06	0.17	0.43	0.00	0	0	0	4	3
18	58039276	rs2229616	C	T	MC4R	Obesity, autosomal dominant	0.00	0.02	0.02	0.02	0.00	0	7	0	0	5
16	67516945	rs5030980	C	T	AGRP	Obesity, late-onset	0.08	0.01	0	0.03	0.00	0	10	0	0	5
3	12475557	rs3856806	C	T	PPARG	Obesity, severe	0.11	0.05	0.21	0.12	0.07	0	8	0	0	5
17	3397702	rs12948217	C	T	ASPA	Canavan disease	0.29	0.23	0.05	0.31	0.55	0	3	0	2	4
5	131676320	rs1050152	C	T	SLC22A4	Celiac disease,Crohn's disease	0.33	0.02	0	0.39	0.34	0	5	0	3	3
14	81575005	rs3783941	C	A	TSHR	Congenital Hypo Thyroidsm	0.76	0.64	0.39	0.67	0.75	7	2	0	0	4
7	117199533	rs213950	G	A	CFTR	Cystic fibrosis	0.28	0.93	0.39	0.44	0.21	3	0	6	0	3
9	111651620	rs3204145	A	T	IKBKAP	Dysautonomia, familial	0.17	0.21	0.29	0.17	0.23	4	0	0	1	2
1	169511755	rs4524	T	C	F5	Factor V	0.26	0.17	0.25	0.25	0.52	0	4	0	4	4
1	169519049	rs6025	C	T	F5	Factor V	0.08	0	0	0.01	0.00	0	5	0	0	2
16	3292874	rs2741918	C	T	MEFV	Familial Mediterranean fever	0.57	0.67	0.61	0.56	0.63	0	3	0	5	4
16	3304463	rs224222	C	T	MEFV	Familial Mediterranean fever	0.20	0.04	0.03	0.28	0.08	0	13	0	2	4
9	97873957	rs4647534	A	G	FANCC	Fanconi anemia, complementation groupC	0.39	0.6	0.63	0.45	0.40	6	0	2	0	4
1	100316589	rs2307130	A	G	AGL	Glycogen storage disease IIIb	0.40	0.22	0.54	0.46	0.25	8	0	3	0	5
8	19819724	rs328	C	G	LPL	HDL cholesterol	0.08	0.06	0.12	0.13	0.29	0	10	6	0	5
8	27373865	rs751141	G	A	EPHX2	Hypercholesterolemia, familial, due to LDLR defect, modifier of	0.05	0.11	0.23	0.09	0.00	0	0	8	0	4
5	42700044	rs6179	A	G	GHR	Hypercholesterolemia, familial, modifier	0.67	0.4	0.82	0.68	0.21	6	0	1	0	4
5	42719239	rs6180	A	C	GHR	Hypercholesterolemia, familial, modifier	0.47	0.38	0.57	0.42	0.26	2	1	0	1	3
11	17417496	rs1800853	G	A	ABCC8	Hyperinsulinemic hypoglycemia, familial, 1	0.02	0.04	0.03	0.01	0.10	1	0	13	0	5
7	113518434	rs1799999	C	A	PPP1R3A	Insulin resistance, severe, digenic	0.16	0.18	0.64	0.12	0.00	0	6	0	0	5
12	112939853	rs3741983	C	T	PTPN11	Insulin resistance, susceptibility to	0.27	0.8	0.84	0.27	0.70	0	5	0	5	4
19	11241915	rs6413504	A	G	LDLR	LDL cholesterol	0.50	0.14	0.3	0.48	0.36	10	0	6	0	5
4	38799710	rs4833095	T	C	TLR1	Leprosy, immunity	0.49	0.88	0.6	0.28	0.15	0	1	0	10	5
1	100672060	rs12021720	T	C	DBT	Maple syrup urine disease, type II	0.89	0.74	0.95	0.92	1.00	0	10	0	0	4
15	72643444	rs117160567	A	C	HEXA	Tay-Sachs disease	0.01	0	0	0.02	0.00	15	0	0	0	4
21	44480616	rs1801181	G	A	CBS	Thrombosis, hyperhomocysteinemic	0.34	0.02	0.57	0.39	0.28	3	0	4	0	3
10	56129066	rs11594958	A	G	PCDH15	Usher syndrome, type 1F	0.25	0.22	0.09	0.22	0.18	6	0	3	0	4
1	68903942	rs12145904	C	T	RPE65	Vitamin A metabolic defect	0.11	0.23	0.36	0.13	0.14	0	5	0	1	2
9	134385599	rs2018621	A	G	POMT1	Walker–Warburg syndrome	0.94	0.72	0.92	0.94	0.83	1	0	8	0	5
14	77746310	rs438931	G	A	POMT2	Walker–Warburg syndrome	0.09	0.13	0.33	0.13	0.00	0	0	10	0	4

Table S3. Modeling Sidon_BA as Levant_N and an ancient population *A*. Sidon_BA can be modelled as a mix of Neolithic Levant 0.484±0.042 and Chalcolithic Iran 0.516±0.042 using a large number of outgroups: Ust_Ishim, Kostenki14, MA1, Han, Papuan, Ami, Chukchi, Karitiana, Mbuti, Switzerland_HG, EHG, WHG, and CHG.

		Mixture Proportions		
**A**	**P-value for rank=1**	**Levant_N**	**A**	**Std. Error**
Iran_ChL	27.89E-02	0.484	0.516	0.042
Iran_HotuIIIb	2.50E-02	0.593	0.407	0.048
Iran_LN	2.45E-02	0.559	0.441	0.044
Iran_recent	1.16E-02	0.309	0.691	0.089
Natufian	0.94E-02	2.33	-1.33	0.376
Iran_N	0.45E-02	0.592	0.408	0.041
Armenia_EBA	0.02E-02	0.528	0.472	0.046
Anatolia_ChL	2.57E-07	0.324	0.676	0.123
Armenia_MLBA	8.78E-11	0.653	0.347	0.052
Europe_MNChL	6.11E-13	1.261	-0.261	0.053
Iberia_BA	3.82E-13	1.375	-0.375	0.107
Armenia_ChL	4.97E-14	0.692	0.308	0.063
Europe_EN	5.73E-15	1.475	-0.475	0.142
SHG	3.50E-16	1.072	-0.072	0.017
Anatolia_N	6.71E-17	1.746	-0.746	0.411
Steppe_IA	4.02E-18	0.891	0.109	0.039
Steppe_EMBA	7.68E-19	0.928	0.072	0.027
Europe_LNBA	6.90E-20	1.053	-0.053	0.04
Steppe_Eneolithic	5.79E-20	0.978	0.022	0.018
Steppe_MLBA	2.71E-20	1.005	-0.005	0.036

Table S4. Counts of Y-chromosome ancestral and derived allele genotypes in the Sidon_BA males

	ERS1790733 observed genotypes	
Haplogroup	Number of ancestral alleles	Number of derived alleles
A00	22	0
A0-T	0	7
A0	17	0
A1	0	4
A1a	8	0
A1b	1	14
BT	0	121
B	1	0
CT	0	73
DE	10	0
CF	0	2
C	2	0
F	0	10
F1	2	0
F3	1	0
GHIJK	0	1
G	70	1
H	8	0
IJK	0	2
IJ	0	2
I	51	1
J	0	31
J1	0	1
J2	1	0
J1a	0	1
J2a	2	0
J2b	2	0
J1a3	1	0
J1a2a	1	0
J1a2b	0	1
J1a2b3	1	0
J1a2b3a	1	0

	ERS1790732 observed genotypes	
Haplogroup	Number of ancestral alleles	Number of derived alleles
A00	26	0
A0-T	0	15
A0	12	0
A1	0	4
A1a	7	0
A1b	1	15
BT	0	171
B	1	0
CT	1	106
DE	13	0
CF	0	1
C	5	0
F	0	17
F1	2	0
F3	1	0
GHIJK	0	1
G	81	1
H	10	0
IJK	0	2
IJ	0	3
I	63	0
J	0	40
J1	1	0
J2	0	1
J1a	1	0
J2a	3	0
J2b	0	2
J2b2a	1	0
J2b2a1	1	0
J2b2a1a	1	0
J2b2a1a1	1	0

Table S5. mtDNA haplogroups frequencies in present-day populations and Sidon_BA

Population (n)	A	B	C	D	F	G	H	HV	I	J1	J2	K	L0	L1	L2	L3	L4	L5	L6	M	N	R	T1	T2	U1	U2	U3	U4	U5	U6	U7	U8	V	W	X	Z3
**Egyptian (100)**							0.20	0.04	0.02	0.03	0.02	0.04		0.04	0.06	0.10	0.01			0.03	0.07	0.04	0.08	0.09	0.01	0.01	0.01		0.01	0.02	0.01	0.01			0.05	
**Ethiopian (124)**								0.03	0.01	0.02		0.02	0.09	0.02	0.13	0.32	0.06	0.04	0.02	0.09	0.06	0.08				0.01		0.01								
**Greek (100)**			0.01				0.44			0.07	0.01	0.02								0.01	0.01	0.04	0.03	0.07	0.02	0.01	0.01	0.01	0.13		0.02	0.01	0.03	0.02	0.03	
**Lebanese (103)**							0.20	0.09	0.03	0.08	0.03	0.08	0.01							0.01	0.08	0.11	0.05	0.06	0.06	0.01	0.03	0.01			0.02	0.03		0.02	0.01	
**CEU (99)**							0.51	0.01	0.01	0.06	0.02	0.03											0.03	0.07		0.02	0.01	0.02	0.13				0.03	0.05		
**Han (103)**	0.07	0.09	0.05	0.22	0.16	0.05						0.01								0.18	0.08	0.09														0.01
**YRI (109)**													0.05	0.16	0.35	0.44	0.01																			
**Sidon_BA (5)**							0.20	0.20				0.20									0.20	0.20														

Table S6: Modelling Lebanese as Sidon_BA and an ancient population *A*. Lebanese can be modelled as a mix of Bronze Age Sidon 0.93±0.018 and a steppe ancestry 0.07±0.018 using outgroups: Ust_Ishim, Kostenki14, MA1, Han, Papuan, Ami, Chukchi, Karitiana, Mbuti, Switzerland_HG, EHG, WHG, and CHG.

A	P-value for rank=1	Mixture Proportions		
		Sidon_BA	A	Std. Error
Steppe_MLBA	9.49E-02	0.924	0.076	0.018
Steppe_EMBA	9.48E-02	0.932	0.068	0.016
Steppe_IA	9.28E-02	0.92	0.08	0.02
Europe_LNBA	5.14E-02	0.927	0.073	0.019
Steppe_Eneolithic	5.14E-02	0.962	0.038	0.011
Iran_LN	3.19E-02	1.16	-0.16	0.063
SHG	3.01E-02	0.972	0.028	0.008
Iberia_BA	1.54E-02	0.934	0.066	0.022
Iran_recent	1.24E-02	0.69	0.31	0.151
Armenia_MLBA	1.04E-02	0.882	0.118	0.041
Armenia_ChL	7.52E-03	0.887	0.113	0.042
Iran_N	5.70E-03	1.104	-0.104	0.055
Natufian	3.93E-03	1.08	-0.08	0.042
Europe_MNChL	2.69E-03	0.962	0.038	0.019
Anatolia_ChL	2.10E-03	0.881	0.119	0.087
Mota	1.64E-03	1.007	-0.007	0.004
Europe_EN	1.51E-03	0.95	0.05	0.033
Iran_ChL	1.15E-03	1.087	-0.087	0.094
Anatolia_N	7.98E-04	0.968	0.032	0.049
Iran_HotuIIIb	7.34E-04	1.023	-0.023	0.068
Armenia_EBA	6.77E-04	0.989	0.011	0.068

Supplemental references

1. Lazaridis, I., Nadel, D., Rollefson, G., Merrett, D.C., Rohland, N., Mallick, S., Fernandes, D., Novak, M., Gamarra, B., Sirak, K., et al. (2016). Genomic insights into the origin of farming in the ancient Near East. Nature 536, 419-424.
2. Haber, M., Gauguier, D., Youhanna, S., Patterson, N., Moorjani, P., Botigue, L.R., Platt, D.E., Matisoo-Smith, E., Soria-Hernanz, D.F., Wells, R.S., et al. (2013). Genome-wide diversity in the levant reveals recent structuring by culture. PLoS genetics 9, e1003316.
3. Renaud, G., Slon, V., Duggan, A.T., and Kelso, J. (2015). Schmutzi: estimation of contamination and endogenous mitochondrial consensus calling for ancient DNA. Genome biology 16, 224.
4. Rasmussen, M., Guo, X., Wang, Y., Lohmueller, K.E., Rasmussen, S., Albrechtsen, A., Skotte, L., Lindgreen, S., Metspalu, M., Jombart, T., et al. (2011). An Aboriginal Australian genome reveals separate human dispersals into Asia. Science 334, 94-98.
